# HIDEEP: a systems approach to predict hormone impacts on drug efficacy based on effect paths

**DOI:** 10.1038/s41598-017-16855-8

**Published:** 2017-11-30

**Authors:** Mijin Kwon, Jinmyung Jung, Hasun Yu, Doheon Lee

**Affiliations:** 1 Department of Bio and Brain Engineering, KAIST, 291 Daehak-ro, Yuseong-gu, Daejeon, Republic of Korea; 2 Bio-Synergy Research Center, 291 Daehak-ro, Yuseong-gu, Daejeon, Republic of Korea; 3 Department of Applied Statistics, College of Economics and Business, The University of Suwon, Bongdam-eup, Hwaseong, Republic of Korea

## Abstract

Experimental evidence has shown that some of the human endogenous hormones significantly affect drug efficacy. Since hormone status varies with individual physiological states, it is essential to understand the interplay of hormones and drugs for precision medicine. Here, we developed an *in silico* method to predict interactions between 283 human endogenous hormones and 590 drugs for 20 diseases including cancers and non-cancer diseases. We extracted hormone effect paths and drug effect paths from a large-scale molecular network that contains protein interactions, transcriptional regulations, and signaling interactions. If two kinds of effect paths for a hormone-drug pair intersect closely, we expect that the influence of the hormone on the drug efficacy is significant. It has been shown that the proposed method correctly distinguishes hormone-drug pairs with known interactions from random pairs in blind experiments. In addition, the method can suggest underlying interaction mechanisms at the molecular level so that it helps us to better understand the interplay of hormones and drugs.

## Introduction

Hormone-drug interactions are crucial for drug treatment, thus sufficient understanding of relations between hormones and drugs is required. Numerous reports have concluded that hormones can change drug efficacy. For example, a group found that stress hormones (e.g. cortisol, norepinephrine, and epinephrine) significantly decrease the apoptotic efficacy of paclitaxel in triple-negative breast cancer cells by affecting DNA damage and cell cycle regulation^1^. In addition, another group observed that men and women with chronic depression showed different responses to treatment of sertraline and imipramine, and further reported that female sex hormones can improve response to sertraline and hinder response to imipramine^2^. However, only small part of hormone-drug pairs among an enormous number of hormones and drugs have been studied so far. Most of the previous studies have focused on only certain types of hormones (especially stress hormones) and drugs (especially cancer-treating drugs). For the better understanding of relationships between hormones and drugs, we need a model that can systematically test all pairwise combinations of them.

Most of the related previous studies have been performed through *in vitro* or *in vivo* based approaches, particularly using cell lines because mainly studied drugs were cancer-treating drugs. For example, in an experiment to compare apoptotic cells with/without glucocorticoid pretreatment before chemotherapy of paclitaxel and doxorubicin respectively in breast cancer cell lines (MCF-7 and MDA-MB-231), it resulted that glucocorticoids constrain apoptosis induced by chemotherapy^3^. *In vitro* or *in vivo* based approaches are accurate and reliable, but they are not appropriate for screening whole pairwise combinations of hormones and drugs because they require high costs. Recently an *in silico* based approach was used for hormone and drug study. For example, *in silico* modeling was performed to uncover how epinephrine affects apoptosis-regulating mechanisms of eight prostate cancer drugs, using ordinary differential equations (ODE)^4^. They found that epinephrine activates anti-apoptotic signaling pathways and eventually decreases the chemotherapeutic efficacy of prostate cancer drugs. Parametric characteristics of quantitative models such as ODE models facilitate accurate network analysis but require optimization of numerous parameters. Thus, the performance of quantitative modeling is limited to small networks that should be determined by prior knowledge. However, the studies of hormones and drugs require comprehensive and large-scale networks because hormones can affect drug efficacy by inducing signaling crosstalk to drug mechanisms of action (MOA) in indirect ways as well as direct ways. For example, epinephrine causes distant signaling crosstalk to MOA of prostate cancer drugs via intermediate molecules^4^. We thus need comprehensive and large-scale networks including MOA of drugs and their neighboring pathways. Large-scale and heterogeneous networks have been frequently utilized to predict interactions between two entities. Some research groups inferred drug-target interactions by using heterogeneous networks consisting of three sub-networks whose edges represent drug-drug chemical structure similarity, target-target sequence similarity, and known drug-target interactions^5,6^. The models showed good performance for drug-target prediction problem, but they are not fit to predict hormone-drug relations (more exactly hormone impacts on drug efficacy). For example, hormones consist of non-steroid hormones such as peptide hormones as well as steroid hormones, which makes it hard to calculate chemical structure similarity between hormones.

Here, we take advantage of large-scale networks to develop a new *in silico* model for screening the interplay of hormones and drugs. We aim to discover that hormones affecting drug efficacy under a specific disease condition. To this end, firstly we construct a biological network by collecting molecular interactions from public databases. Secondly, two kinds of paths for a hormone-drug pair are inferred: (1) drug effect paths and (2) hormone effect paths. Drug effect paths (DEPs) are defined as all possible shortest paths from each drug target to the nearest disease gene, which implies drug MOA. Hormone effect paths (HEPs) are defined as the shortest paths from hormone receptors to the nearest molecule of the DEPs, and HEPs possibly induce signaling crosstalk to drug MOA. Lastly, a scoring function is defined under the main assumption that a hormone whose receptors are closer to DEPs of a drug has higher potential to affect the drug efficacy than distant hormones. High-ranked but yet unknown hormone-drug pairs are suggested as candidates, and they are supported by their underlying mechanisms of interference inferred by the proposed method. This approach is termed as a prediction of Hormone Impact on Drug Efficacy based on Effect Paths (HIDEEP). We hope that this large-scale network-based approach will improve our understanding of crosstalks between a hormone and a drug.

## Results

### Data collection

A biological network consists of molecular interactions collected from three public databases: BioGRID^7^, KEGG pathways^8^, TRANSFAC^9^ (see Materials and methods). Total 192,232 of molecular interactions are collected, including 189,417 gene-gene interactions, 1,198 gene-compound interactions, and 1,617 compound-compound interactions between 16,744 genes and 1,487 compounds. We collect following data from public databases: 1) human endogenous hormones and their receptors from EndoNet^10^, 2) drug-disease associations and disease-gene associations from comparative toxicogenomics database (CTD)^11^, 3) drug-target associations from DrugBank^12^ (see Materials and methods). Hormones, drugs, and diseases respectively should have at least one receptor, target, and disease gene. As a result, 283 human hormones, 4,781 drugs, and 139 diseases are finally extracted.

### Disease selection

Twenty diseases are selected under three criteria for case studies: 1) the number of gold standard samples is three or more, 2) the number of disease genes is one or more, 3) various disease types are considered (see Materials and methods). Among 4,881 diseases with one or more disease genes, 139 diseases with three or more gold standard samples are filtered. Highly ranked ones out of 139 diseases based on the number of gold standard samples are non-cancer disease types. Thus, fifteen high-ranked non-cancer diseases and extra five cancer diseases are determined as case study diseases. In this way, we choose final twenty diseases, avoiding bias to any certain disease type. Table 1 shows the selected twenty diseases and their number of gold standard samples, disease genes, and drugs.

**Table 1:** The number of gold standard samples, drugs, and disease genes of selected twenty diseases.

No.	Disease	# of gold standard samples	# of drugs	# of disease genes
1	Hypertension	38	199	153
2	Tachycardia	25	88	16
3	Myocardial Infarction	24	88	70
4	Angina Pectoris	23	50	1
5	Diabetes Mellitus, Type 2	22	29	117
6	Atrial Fibrillation	22	42	19
7	Heart Failure	19	86	70
8	Hypotension	17	90	46
9	Seizures	15	189	79
10	Hyperalgesia	15	110	60
11	Hyperglycemia	12	33	18
12	Postoperative Complications	11	55	3
13	Diabetic Nephropathies	11	33	29
14	Venous Thromboembolism	9	6	8
15	Acute Kidney Injury	7	47	55
16	Breast Neoplasms	6	97	404
17	Colonic Neoplasms	6	48	118
18	Ovarian Neoplasms	4	39	77
19	Lung Neoplasms	4	47	167
20	Prostatic Neoplasms	4	70	450

### Drug effect paths

Drug effect paths (DEPs), which imply drug MOA, are defined as the shortest paths from each drug target to the nearest disease-causing gene (see Materials and methods). The biological network we construct does not include drugs as entities, thus DEPs start vicariously from drug targets. The length of each DEP is calculated by distance from a target to a disease gene. For example, the path length of a DEP consisting of three molecules becomes two. Drugs ultimately aim to affect disease genes either by physically bindings to them or by signal transduction via intermediate molecules. In the case of a drug physically binding to a disease gene as a drug target, the length of DEPs becomes zero. A drug may have different DEPs for different disease treatment because DEPs of a drug are determined by disease genes of each disease.

### Hormone effect paths

Hormone effect paths (HEPs), which can cause signaling crosstalk on DEPs, are defined as the shortest paths from hormone receptors to molecules of DEPs (see Materials and methods). The biological network we construct does not include hormones as entities, thus HEPs start vicariously from hormone receptors. A hormone has one or more receptors, and DEPs of a drug consist of one or more molecules. A hormone-drug pair, thus, has receptor-molecule pairwise combinations and each receptor-molecule pair can have the different shortest paths with a different length. Thus, HEPs become the very shortest paths among the all possible shortest paths. HEPs may impact on upstream (e.g. drug target), downstream (e.g. disease gene), or intermediate molecules of DEPs, which results in signaling crosstalk on DEPs.

### Scoring function

For a hormone-drug pair, a potential impact that the hormone has an intervention on the drug efficacy is evaluated by a scoring function defined under the following assumptions: 1) hormones whose receptors are closer to DEPs (i.e. a hormone with shorter HEPs) have a higher potential to affect the efficacy of drugs, 2) the more receptors of a hormone are involved in HEPs, the more the hormone affects the efficacy of the drug, 3) the more molecules of DEPs are involved in HEPs, the more the hormone affects the efficacy of the drug. For example, although two hormones A and B have the same number and length of HEPs for a drug, hormone A which has two receptors involved in HEPs is more likely to have impacts on the efficacy of the drug than hormone B which has only one receptor involved in HEPs. Likewise, although two hormones C and D have the same number and length of HEPs for a drug, hormone C which has two molecules of DEPs involved in HEPs is more likely to have impacts on the efficacy of the drug than hormone D which has only one molecule of DEPs involved in HEPs. Given *h*, a hormone, *d*, a drug, *R*(*h*), the set of hormone receptors, *M*(*d*), the set of molecules of DEPs, *n*(*S*), the number of distinct start nodes (i.e. receptors involved in HEPs), *n*(*E*), the number of distinct end nodes (i.e. molecules of DEPs involved in HEPs), we define a potential impact *i*.as equation (1).

1 $$i(h,d)={\alpha }^{-{\min }_{r\in R,m\in M(d)}d(r,m)}\times n(S)\times n(E)$$

where *d*(*r,m*), the shortest path length from receptor *r* to molecule *m* and *α*, a decay constant. Thus, ${\min }_{r\in R(h),m\in M(d)}d(r,m)$ means a length of HEPs. When there is no HEPs for hormone *h* and drug *d*, the length of HEPs is represented by infinite and *i*(*h, d*) converges to zero. A hormone-drug pair whose *n*(*S*) or *n*(*E*) is higher has a stronger potential impact *i*.

### Model evaluation

For model evaluation, unlabeled hormone-drug pairs for each disease are randomly sampled from the human hormone set and the corresponding disease-treating drug set. A dataset for each disease consists of a gold standard set and an unlabeled set. In order to cover different areas of the search space, each disease has five datasets that consist of five different unlabeled sets sampled by multiple permutations. In addition, to show results for different sizes of unlabeled sets, we determine the five different sizes of unlabeled sets (i.e. 1, 3, 5, 7, and 10 times of the size of the corresponding gold standard sets) (Supplementary Table 1). Thus, each disease has five datasets per size and totally 25 datasets for the five sizes.

**Figure 1:**
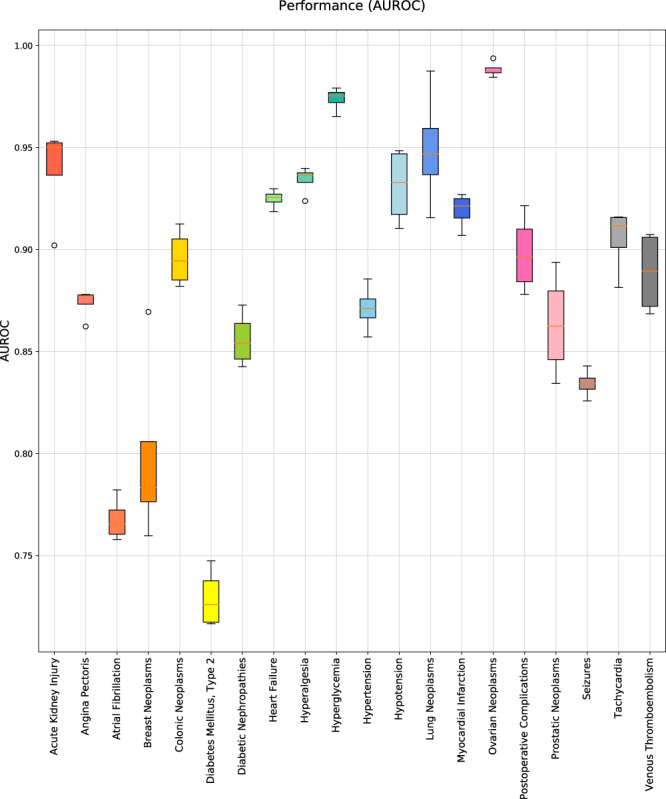
Performance evaluation for five datasets whose unlabeled sets are 10 times of the corresponding gold standard set. We evaluate performance for each disease using five datasets whose unlabeled sets are ‘10 times’ of the gold standard set. The area under the receiver operating characteristic (AUROC) was measured for performance evaluation. Here, α, the decay constant, of the scoring function is 8.

We analyze DEPs and HEPs for every hormone-drug pairs of gold standard sets and unlabeled sets. Every hormone-drug pair is scored by the scoring function. The area under the receiver operating characteristic curve (AUROC) is used to evaluate how well this model distinguishes known hormone-drug pairs from random hormone-drug pairs. In order to calculate AUROC, we draw a Receiver operating characteristic (ROC) curve, whose x-axis is the false positive rate (1-specificity) and y-axis is the true positive rate (sensitivity) while varying threshold scores. In order to optimize a decay constant, *α*, we, first of all, apply HIDEEP to datasets with ‘10 times’ size and evaluate AUROC performance while varying *α* from two to ten. The performance continuously increases as α increases, but it reaches a saturation level at *α* = 8 (AUROC 0.89) (Supplementary Fig. 1). Thus, *α* is determined as eight for model evaluation and performance comparison in this study. For each of the five different sizes, HIDEEP shows the same performance with AUROC 0.89 on average for 20 diseases although there are slight differences in individual diseases depending on the sizes (Table 2). Figure 1 shows AUROC performances for five datasets with size ‘10 times’ for each disease. ROC curves for 20 diseases per data size are also shown in Supplementary Figs 2, 3, 4, 5, and 6. We additionally evaluate model performance using the area under the precision-recall curve (AUPR). The average AUPR for overall 20 diseases is 0.89, 0.77, 0.66, 0.60, and 0.55 respectively for five unlabeled set sizes (1, 3, 5, 7, and 10 times of gold standard sets). Precision-recall curves for 20 diseases per data size are also shown in Supplementary Figs 7, 8, 9, 10, and 11. The every AUPR is over 0.77 or 0.5 for datasets with size ‘1 time’ or size ‘3 times’.

**Table 2:** Performance evaluation of the HIDEEP per the size of unlabeled datasets (AUROC).

No.	Disease	AUROC				
		1 times	3 times	5 times	7 times	10 times
1	Acute Kidney Injury	0.92	0.95	0.95	0.96	0.94
2	Angina Pectoris	0.87	0.87	0.87	0.87	0.87
3	Atrial Fibrillation	0.77	0.76	0.76	0.78	0.77
4	Breast Neoplasms	0.81	0.80	0.80	0.76	0.79
5	Colonic Neoplasms	0.89	0.91	0.90	0.87	0.90
6	Diabetes Mellitus, Type 2	0.76	0.73	0.76	0.74	0.74
7	Diabetic Nephropathies	0.84	0.87	0.88	0.89	0.86
8	Heart Failure	0.95	0.94	0.92	0.93	0.93
9	Hyperalgesia	0.91	0.95	0.93	0.94	0.93
10	Hyperglycemia	0.96	0.98	0.98	0.98	0.97
11	Hypertension	0.85	0.87	0.89	0.88	0.87
12	Hypotension	0.92	0.92	0.91	0.91	0.93
13	Lung Neoplasms	0.89	0.93	0.92	0.95	0.95
14	Myocardial Infarction	0.91	0.91	0.91	0.90	0.92
15	Ovarian Neoplasms	0.94	0.98	0.97	0.96	0.98
16	Postoperative Complications	0.92	0.94	0.92	0.92	0.90
17	Prostatic Neoplasms	0.93	0.86	0.85	0.85	0.86
18	Seizures	0.84	0.83	0.83	0.82	0.83
19	Tachycardia	0.93	0.90	0.91	0.90	0.91
20	Venous Thromboembolism	0.91	0.93	0.90	0.91	0.89
	**Average**	**0.89**	**0.89**	**0.89**	**0.89**	**0.89**

**Figure 2:**
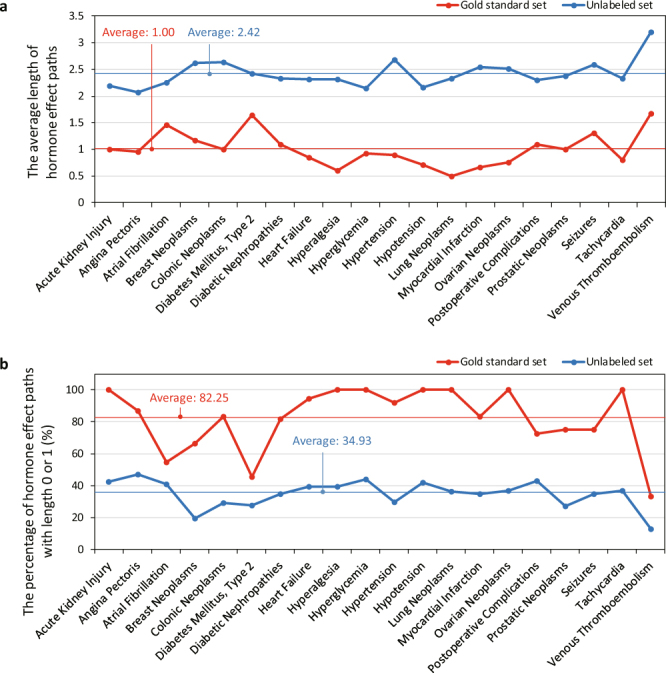
Comparison of hormone effect paths (HEP) lengths of gold standard sets and unlabeled sets. (**a**) The average length of HEPs of gold standard sets (red) and unlabeled sets (blue). (**b**) The percentage (%) of hormone-drug pairs, whose length of HEPs is zero or one, of gold standard sets (red) and Funlabeled sets (blue).

In order to validate the assumption that hormones whose receptors are closer to DEPs have a higher potential to affect the efficacy of drugs, we test how significantly short HEPs of gold standard samples are in comparison to HEPs of unlabeled samples. To this end, we analyze distributions of the average HEP lengths of a gold standard set and five unlabeled sets with ‘10 times’ size for each disease (Supplementary Fig. 12). A two-sample multivariate t-test is used to estimate the significance of the difference between the average HEP lengths of two types of sample sets for a disease, and 18 out of 20 diseases result in less than p-value 0.01 (Supplementary Table 2). The average HEP lengths of a gold standard set and five unlabeled sets with ’10 times’ size for each disease are measured, and those for overall twenty diseases are on average 1.00 and 2.42 respectively (Fig. 2a). The average HEP length of gold standard samples is less than that of unlabeled samples in every disease. In addition, we compare the percentage of hormone-drug pairs whose HEP lengths are zero or one for a gold standard set and unlabeled sets for each disease (Fig. 2b). The percentage of a gold standard set exceeds that of unlabeled sets in every disease. Compared to 34.93% of unlabeled sets, 82.25% of gold standard sets on average for twenty diseases have HEPs with length either zero or one.

We further evaluate whether the proposed method, HIDEEP, still robustly performs when taking out some information from the network. There are unique 451 receptors for the 283 hormones: 428 receptors for only a single hormone, 17 receptors for two hormones, two receptors for three hormones, one receptor for four hormones, one receptor for seven hormones, and two receptors for eight hormones. We select top six receptors that have the most number of interacting hormones under the assumption that a receptor interacting with more hormones is more likely to be critical for the performance of the model: LTB4R2 and CYSLTR1 (eight hormones), LTB4R (seven hormones), NR3C1 (four hormones), ADRA2A and CRHR2 (three hormones). We reconstruct eight different networks where each of the top six receptors is removed (case 1–6), a network where the top three receptors are removed (case 7), and a network where the all top six receptors are all removed (case 8). The average AUROC value for 20 diseases is either 0.89 or 0.90 in every case, which shows that there is no big difference from the original case performing AUROC 0.89 for overall 20 diseases in every dataset size (1, 3, 5, 7, and 10 times of gold standard sets). These results indicate that HIDEEP seems to be robust even when part of the information is hidden.

### Performance comparison

**Figure 3:**
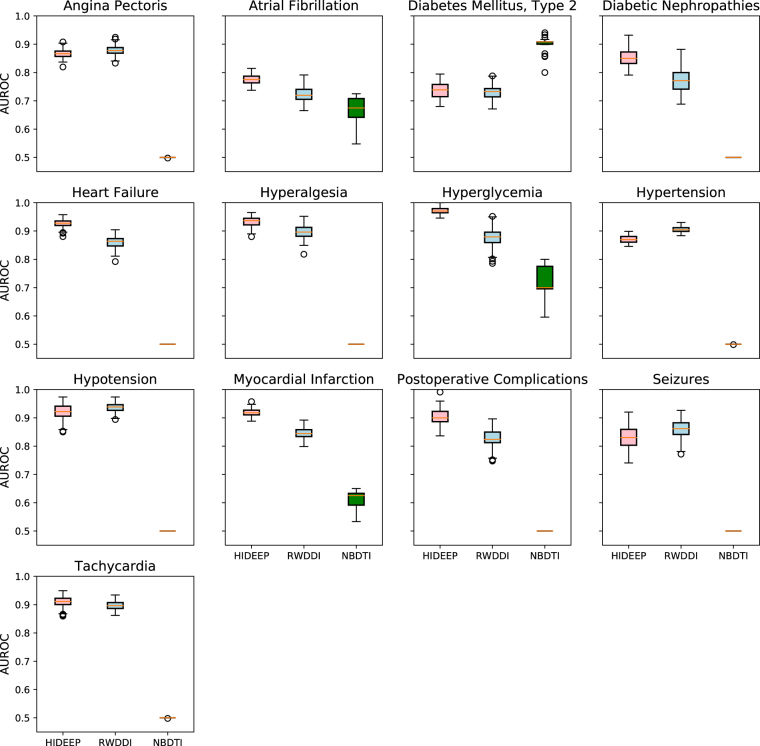
Performance comparison with two previous methods. The performance of HIDEEP (pink), RWDDI (light blue) and NBDTI (green) for 13 diseases was evaluated by the area under the receiver operating characteristic curve (AUROC). For this performance comparison, we adopt an evaluation strategy, 30 times of 10-fold cross validation used to evaluate NBDTI in the original study. HIDEEP, RWDDI and NBDTI are applied to five datasets consisting of a gold standard set and five unlabeled sets with ‘10 times’ for 13 diseases with 10 or more positive samples. Thus, each box includes 150 AUROC values for five datasets.

We compare the proposed method, HIDEEP, with a state-of-the-art method which is closely related to HIDEEP despite the fact that it was published to handle a slightly different problem, prediction of drug-drug interactions^13^. This study predicts pharmacodynamic drug-drug interactions through signaling propagation interference using the random walk with restart algorithm on molecular networks (shortly called RWDDI). It uses network features only to define a scoring function and represents drugs with their receptor proteins, which makes it available to apply this model to our problem. We score gold standard sets and unlabeled sets with ‘10 times’ size for 20 diseases by RWDDI. Here, restarting probability of the random walker at each time step, *r*, is determined to 0.7 as it was in the original study. The average, minimum and maximum AUROC values of HIDEEP are respectively 0.89 (all diseases), 0.74 (diabetes mellitus, type 2) and 0.98 (lung neoplasms), with standard deviation 0.0653 (Fig. 3). Whereas, the average, minimum and maximum AUROC values of RWDDI are respectively 0.84 (all diseases), 0.59 (breast neoplasms) and 0.97 (prostatic neoplasms), with standard deviation 0.0855 (Supplementary Fig. 13). Additionally, the average AUPR of RWDDI for overall 20 diseases is 0.44 while that of HIDEEP is 0.55. These results show that HIDEEP is more robust for disease types compared to RWDDI.

HIDEEP is compared with one other closely related method, prediction of drug-target interactions (DTI), although it was developed to solve a slightly different problem^14^. This study implemented a bipartite drug-target network-based DTI inference (shortly called NBDTI). NBDTI requires only information of known DTIs to predict new DTIs, which makes it available to apply this model to infer hormone-drug interactions. For performance comparison, we adopt the same evaluation strategy, 30 times of 10-fold cross-validation used in NBDTI study. The 13 out of 20 diseases have ten or more positive samples, and the 13 diseases are selected. For cross-validation setup, we firstly generate ten folds using one out of five datasets that consist of gold standard sets and unlabeled sets with ‘10 times’ size. Each of the ten folds has an identical ratio of gold standard samples and unlabeled samples. One fold is taken for testing a model while the other nine folds are used for training the model if needed. This procedure is implemented repeatedly ten times, switching a target test fold. The previous sequence from generating ten folds to switching a test fold ten times is iterated 30 times. The previous whole process is identically applied to the all five datasets. The three methods (NBDTI, RWDDI, and HIDEEP) are tested with the same test folds for a fair comparison. Based on this cross-validation strategy, the average AUROC for 13 diseases is measured. HIDEEP shows the best performance at *α* = 8 among values from two to ten, which is consistent with the value of the alpha previously tuned by using all datasets (Supplementary Table 3). Figure 3 shows the result of performance comparison. Firstly, the average, minimum and maximum AUROC values of NBDTI are respectively 0.57 (all diseases), 0.5 (hypertension, tachycardia, angina pectoris, heart failure, hypotension, seizures, hyperalgesia, postoperative complications, diabetic nephropathies) and 0.91 (diabetes mellitus, type 2). The average, minimum and maximum AUROC values of RWDDI are respectively 0.84 (all diseases), 0.72 (atrial fibrillation) and 0.94 (hypotension). Whereas, the average, minimum and maximum AUROC values of HIDEEP at *α* = 8 are respectively 0.88 (all diseases), 0.74 (diabetes mellitus, type 2), 0.97 (hyperglycemia). The standard deviations of NBDTI, RWDDI, and HIDEEP are respectively 0.1216, 0.0641, and 0.0658. The average AUPRs of RWDDI and NBDTI for overall 13 diseases are respectively 0.52 and 0.60 whereas that of HIDEEP is 0.58. NBDTI shows random performance 0.5 for the nine of thirteen diseases. We figure out that NBDTI is a known drug-target (or hormone-drug) interaction-based approach and its characteristic is that only drugs (or hormones) with at least one target (or drug) can be inferred to interaction with other targets (or drugs). That is, if either a hormone or a drug in a test pair is not included in the bipartite network, this pair is unavailable to be tested by NBDTI and has a score, zero. Unlike drug-target interactions, not many hormone-drug interactions have been revealed yet, and NBDIT is not appropriate to predict a problem that does not have sufficient positive samples such as hormone impacts on drug efficacy.

### Previous work reproduction

**Figure 4:**
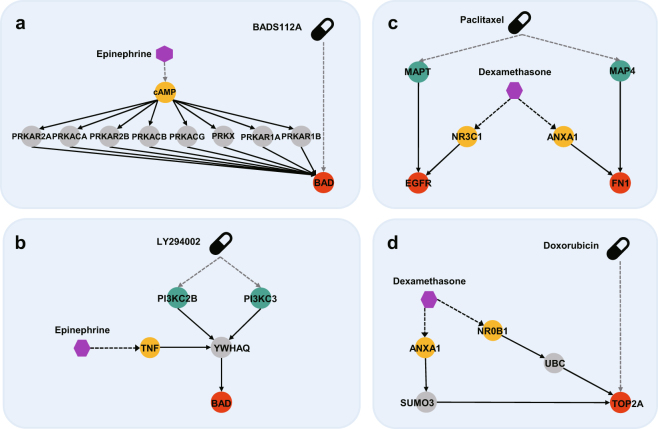
Underlying mechanism analysis of previous works through HIDEEP. Each node type means drug (black), hormone (purple), drug target (green), disease gene (red), hormone target (yellow), intermediate molecule (gray). HIDEEP analyze crosstalk mechanisms of known hormone-drug pairs discovered by previous studies. (**a**) Epinephrine-BADS112A (prostate cancer). (**b**) Epinephrine-LY294002 (prostate cancer). (**c**) Dexamethasone-paclitaxel (breast cancer). (**d**) Dexamethasone-doxorubicin (breast cancer).

One previous study quantitatively modeled MOA of epinephrine and eight prostate cancer drugs by using ODE^4^. This ODE-based model could describe how epinephrine impacts on the drug actions. The eight drugs are inhibitors for molecules of signaling pathways which activates anti-apoptosis in prostate cancer. Because those inhibitors are not included in the drug list in this study, we further test the combinations of epinephrine and two inhibitors (BADS112A, LY294002) whose efficacy is the highest among eight inhibitors. First, as for the epinephrine-BADS112A pair, they figured out that while BADS112A inhibits BAD whose phosphorylated proteins have an anti-apoptotic function, epinephrine has the reverse action of BADS112A in the following way: epinephrine activates cAMP, cAMP activates PKA, and PKA activates BAD. HIDEEP results that BADS112A inhibits BAD and epinephrine has HEPs such as cAMP → PKA family → BAD (Fig. 4a). The underlying mechanisms of epinephrine and BADS112A are exactly identical to the findings of the previous study. Second, as for the epinephrine-LY294002 pair, the previous study figured out that while LY294002 inhibits PI3K which activates BAD, epinephrine has the reverse action of LY294002 in the following ways: epinephrine activates cAMP, cAMP activates PKA, and PKA activates BAD. HIDEEP results that LY294002 has DEPs such as PI3K family → YWHAQ → BAD and epinephrine has a HEP such as TNF → YWHAQ (Fig. 4b). They used indirect association between PI3K and BAD due to lack of molecular interaction information, but HIDEEP reveals more in detail that PI3K family has signal transduction to BAD via ‘YWHAQ’^15,16^. In addition, HIDEEP infers that downstream signaling path of epinephrine induces signaling crosstalk on YWHAQ of DEPs of LY294002z and related findings are observed in previous studies^17,18^.

We also examine hormone-drug pairs tested in the previous breast cancer study which is one of *in vitro* based study and which has a limitation to account for underlying mechanisms of interference^3^. This study reported that dexamethasone decreases the apoptotic efficacy of both paclitaxel and doxorubicin treating breast cancer. HIDEEP reveals that paclitaxel has DEPs such as MAPT → EGFR and MAP4 → FN1 and dexamethasone impacts on EGFR and FN1 via NR3C1 and ANXA1 respectively, which implies changes in the efficacy of paclitaxel^19,20^ (Fig. 4c). Furthermore, HIDEEP reveals that doxorubicin directly targets TOP2A, one of breast cancer-causing genes, and dexamethasone has impacts on TOP2A through HEPs such as ANXA1 → SUMO3 → TOP2A and NR0B1 → UBC → TOP2A (Fig. 4d). These uncovered molecular relations of the DEPs and HEPs have been observed in previous studies^21–24^.

### Network analysis for top three diseases

For overall five data sizes, HIDEEP averagely best performs for hyperglycemia (AUROC 0.98), ovarian neoplasms (AUROC 0.97), and acute kidney injury (AUROC 0.95) among twenty diseases, and we implement network analysis for hormone-drug pairs on high ranks for the three diseases in order to help us better understand the interplay between hormones and drugs. To this end, for each disease we select maximum three hormone-drug pairs satisfying following criteria: 1) pairs within top ten in each dataset, 2) pairs with over score ‘1.0’, and 3) unlabeled pairs.

**Figure 5:**
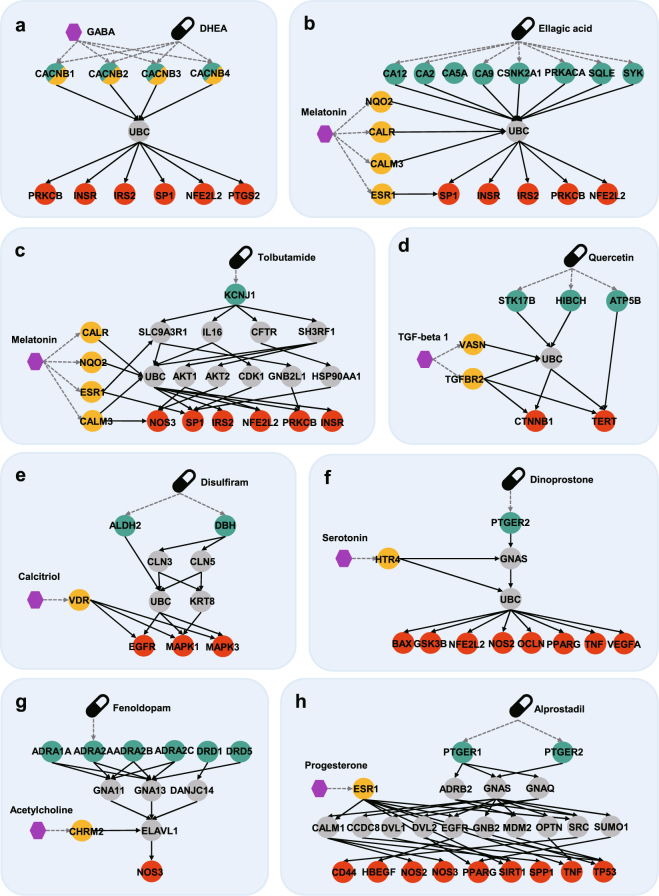
Underlying mechanisms of hormone-drug pairs with high scores for top 3 diseases, hyperglycemia, ovarian neoplasms and acute kidney injury. Each node type means drug (black), hormone (purple), drug target (green), disease gene (red), hormone target (yellow), intermediate molecule (gray). Each figure shows how a hormone causes crosstalk to drug MOA using drug effect paths and hormone effect paths analyzed by HIDEEP. (**a**–**c**) Three figures are for hyperglycemia. (**d**,**e**) Two figures are for ovarian neoplasms. (**f**–**h**) Three figures are for acute kidney injury.

First, hyperglycemia has five hormone-drug pairs satisfying above criteria, and we analyze underlying mechanisms of the three pairs with high scores through HIDEEP to support the evidence. Dehydroepiandrosterone (DHEA) has DEPs from 14 drug targets (i.e. BABRA1, BABRA2, BABRA3, BABR4, BABR5, BABR6, BABRB1, BABR2, BABRD, BABRG1, BABRG2, BABRG3, BABRP, and BABRQ) to eleven disease genes (i.e. SP1, PRKCB, NF32L2, INS, INSR, IRS2, NOS3, PTGS2, HMGA1, GCK, and SIM1) via intermediate molecules. Whereas, gamma-aminobutyric acid (GABA) physically binds to the 14 targets as well, which means that DHEA and GABA share the same targets and receptors. Figure 5a shows some crosstalk mechanisms whose DEPs are the shortest. Ellagic acid has DEPs from 15 drug targets (i.e. CA1, CA12, CA14, CA2, CA3, CA4, CA5A, CA5, CA9, CSNK2A1, PRKACA, PRKCA, SQLE, and SYK) to 13 disease genes (i.e. GCK, HMGA1, HSD11B1, INS, INSR, IRS2, LEPR, NFE2L2, NOS3, PRKCB, PTGS2, SIM1, and SP1) via intermediate molecules. Whereas, melatonin has HEPs with length one from six receptors (i.e. CALM3, CALR, ESR1, MTNR1A, MTNR1B, and NQO2) to 27 molecules of DEPs. Figure 5b shows some crosstalk mechanisms whose DEPs are the shortest. Tolbutamide has DEPs from two drug targets (i.e. ABCC8, KCNJ1) to eleven disease genes (i.e. HMGA1, HSD11B1, INS, INSR, IRS2. NF32L2, NOS3, PRKCB, PTGS2, and SP1) via intermediate molecules. Whereas, melatonin has HEPs with length one from four receptors (i.e. CALMC, CALR, ESR1, and NQO2) to 14 molecules of DEPs. Figure 5c shows some crosstalk mechanisms whose DEPs are the shortest. Melatonin has been reported to suppress hyperglycemia, which implies that it can help therapeutic influence of ellagic acid and tolbutamide^25^.

Second, ovarian neoplasm has two hormone-drug pairs satisfying above criteria. Quercetin has DEPs from three drug targets (i.e. ATP5B, HIBCH, and STK17B) to 24 disease genes (i.e. AKT1, BIRC5, BRCA2, CCNE1, CDH1, CDKN1B, CTNNB1, EDNRA, EGFR, ERBB2, FASN, HDAC6, IL6ST, MAPK1, MAPK3, MET, MLH1, NR5A1, SKP2, SOD1, STAT3, TERT, XIAP, and YAP1) via intermediate molecules. Whereas, transforming growth factor beta 1 (TGF-beta1) has HEPs with length one from two receptors (i.e. VASN and TGFBR2) to three molecules of DEPs. Figure 5d shows some particular crosstalk mechanisms whose DEPs are the shortest or crosstalk mechanisms that TGF-beta 1 affects greater than two molecules of each DEP. TGF-beta1 has been reported to modulate ovarian cancer invasion through upregulation of CAF-derived versican, which implies that it may have interference on the therapeutic efficacy of quercetin^26^. Disulfiram has DEPs from two drug targets (i.e. ALDH2 and DBH) to three disease genes (i.e. EGFR, MAPK1, and MAPK3) via intermediate molecules. Whereas, calcitriol has HEPs with length one from one receptor (VDR) to three molecules of DEPs. Figure 5e shows all possible crosstalk mechanisms inferred by HIDEEP. Calcitriol has been observed to inhibit the proliferation of ovarian cancer cells, which implies that it can help therapeutic influence of disulfiram^27^.

Third, acute kidney injury has five hormone-drug pairs satisfying above criteria, and we select three pairs with high scores for mechanism analysis. Dinoprostone has DEPs from three drug targets (i.e. PTGER1, PTGER2, and PTGER4) to 18 disease genes (i.e. ALB, BAX, CCR5, CD44, GSK3B, HBEGF, HSPA1A, NFE2L2, NOS2, NOS3, OCLN, PPARG, RAPGER3, SIRT1, SPP1, TNF, TP53, and VEGFA) via intermediate molecules. Whereas, serotonin has HEPs with length one from 14 receptors (i.e. ADRA2A, ADRA2B, HTR1A, HTR1B, HTR1D, HTR1E, HTR1F, HTR2A, HTR2B, HTR2C, HTR4, HTR5A, HT6, and HTR7) to nine molecules of DEPs. Figure 5f shows the particular crosstalk mechanisms that serotonin’s HEPs affect more than two molecules of each DEP. It was observed that serotonin reuptake inhibition causes kidney vasoconstriction with resultant hypoperfusion. In other words, serotonin may have assistant influence for therapeutic efficacy of dinoprostone by preventing or treating acute kidney injury^28^. Fenoldopam has DEPs from seven drug targets (i.e. ADRA1A, ADRA1D, ADRA2A, ADRA2B, ADRA2C, DRD1, and DRD5) to 18 disease genes (i.e. ALB, BAX, CCR5, CD44, GSK3B, HB3GF, HSPA1A, NFE2L2, NOS2, NOS3, OCLN, PPARG, RAPGEF3, SIRT1, SPP1, TNF, TP53, and VEGFA) via intermediate molecules. Whereas, acetylcholine has HEPs with length one from five receptors (i.e. CHRM2, CHRM3, CHRM4, CHRM5, and CHRNB1) to eleven molecules of DEPs. Figure 5g shows some particular crosstalk mechanisms whose DEPs include ELAVL1, one of the intermediate molecules. It was observed that acetylcholine receptor agonist attenuates septic acute kidney injury, which implies that acetylcholine can help therapeutic influence of fenoldopam by suppressing inflammation^29^. Alprostadil has DEPs from two drug targets (i.e. PTGER1 and PTGER2) to 15 disease genes (i.e. BAX, CD44, GSK3B, HBEGF, NFE2L2, NOS2, NOS3, OCLN, PPARG, RAPGEF3, SIRT1, SPP1, TNF, TP53, and VEGFA) via intermediate molecules. Whereas, progesterone has HEPs with length one from four receptors (i.e. ESR1, NR3C2, OPRK1, and PGR) to eleven molecules of DEPs. Figure 5h shows some particular crosstalk mechanisms involving ESR1 which has the most number of HEPs among all receptors. It was observed that progesterone participates in kidney electrolyte balance whose abnormality can cause acute kidney injury, and this observation implies that progesterone can change the therapeutic efficacy of alprostadil^30^.

## Discussion

We developed an *in silico* model called as HIDEEP that predicts hormone-drug pairs whose hormones have potential impacts on drug actions and eventually can change drug efficacy. To this end, we took advantage of comprehensive and large-scale molecular networks in consideration of that a hormone can cause signaling crosstalk to MOA of a drug in direct or indirect (distant) way. HIDEEP has following four characteristics. First, it is not confined to specific disease types but can be applied to any disease which has one or more disease genes. Second, it can be applied to any hormone-drug pair whose hormone receptors and drug targets are known. Third, it gives not simple interactions (e.g. hormone A and drug B interact each other) but intimately directional relations of hormone-drug pairs (e.g. hormone A has an impact on drug B efficacy). Last, it shows underlying mechanisms of candidate hormone-drug pairs and helps to understand how a hormone induces signaling crosstalk to drug MOA and results in changes in drug efficacy.

The average HEP lengths of known hormone-drug pairs exceed those of unlabeled pairs in all twenty diseases, supporting the assumption that hormones have the higher potential to affect the efficacy of drugs if their receptors are closer to DEPs of the drugs. In addition, HIDEEP performs AUROC 0.89 on average for diverse twenty diseases and this result implies that HIDEEP can be widely applied to various kinds of other diseases. The performance comparison with a state-of-the-art method (i.e. RWDDI) and a closely related method (i.e. NBDTI) shows that HIDEEP averagely better performs for overall diseases with high AUROC value and small variance than RWDDI and NBDTI. It implies that the HIDEEP is more robust for disease types compared to the two methods. In addition, HIDEEP uses paths to predict hormone impacts on drug efficacy, thus it can elucidate hormones’ signaling crosstalk mechanism.

Hormones are affected by numerous factors (e.g. a disease, drug treatment, growth, weight change, aging, dietary, or psychological stress), which results in different hormone levels in individuals. As sertraline was determined as a more appropriate antidepressant rather than imipramine for a depressed woman by taking account into the sex hormones^2^, individual hormone difference should be considered for effective drug treatment. Thus, precision medicine can be accelerated by considering hormone impacts on drug efficacy. HIDEEP can serve as a first-step computational approach for high-throughput identification of signaling crosstalks between hormones and drugs and also can give new insights into better precision medicine. Due to limited known hormone-drug pairs whose hormones impact on the efficacy of the drugs, the sizes of gold standard sets used in this study are not sufficiently large. More studies about hormone-drug association are required and hormone-drug pairs with high scores graded by HIDEEP can be suggested as candidates for future *in vitro* or *in vivo* experiments. Additionally, in the future, we plan to challenge weighted networks that can make it feasible for a model to come closer to the real biological system and improve the predictive power.

## Methods

### Integration of massive molecular interactions

We collect molecular interactions from three public databases: BioGRID^7^ (protein-protein interactions), KEGG pathways^8^ (signaling interactions, gene regulatory interactions, and protein-protein interactions), and TRANSFAC^9^ (gene regulatory interactions). In order to integrate heterogeneous data from the three different databases, we use reference identifiers as follows: Entrez identifiers for genes^31^ and KEGG compound identifiers^8^ for chemical compounds. Proteins are represented by their encoding genes, thus each entity of the molecular interactions is either a gene (protein) or a compound.

Interactions from KEGG pathways are collected from KEGG Markup Language (KGML) files using KEGGgraph R package^32^. Interactions from KEGG pathways have sixteen relation types whose entities are genes (proteins) or compounds. We filter out particular relation types if they are metabolic reactions, if the number of relations is too small, or if the meaning or direction of relations is not clear. Interactions for eight relations types, therefore, are extracted (i.e. activation, inhibition, expression, repression, phosphorylation, dephosphorylation, binding/association, and dissociation). Gene regulatory interactions are collected from TRANSFAC as well as KEGG pathways.

Protein-protein interactions facilitate rich network analysis with massive molecular interaction data. Physical interactions are extracted from BioGRID. In order to construct a reliable network, we extract only physical protein interactions which have interactions types such as direct interaction, physical association, and co-localization. For network analysis, protein-protein interactions are considered as bidirectional while the other signaling interactions and gene regulatory interactions are considered as unidirectional.

### Arrangement of hormones, drugs, and disease genes

EndoNet provides physical interactions between human hormones and their receptor proteins. DrugBank provides physical interactions between drugs and their target proteins. We extract hormones that have one or more receptors and drugs which have one or more targets. CTD provides two relation types (‘marker/mechanism’ and ‘therapeutic’) for disease-gene associations. ‘Marker/mechanism’ means that a gene is a biomarker of a disease or play a role in the etiology of disease on the other hand ‘therapeutic’ means that a gene is a therapeutic target for disease treatment^11^. In order to consider etiology associations, only disease-gene associations with ‘marker/mechanism’ are extracted.

### Arrangement of gold standard sets

There has been no database or article yet which provides lists of hormone-drug pairs whose hormones affect drug efficacy. In order to define gold standard sets for each disease, we determine to extract hormone-drug interactions from drug-drug interactions. DrugBank provides drug-drug interactions and their detailed descriptions which include directional relations between drugs (e.g. trastuzumab-doxorubicin: trastuzumab may increase the cardiotoxic activities of doxorubicin). Therefore, we firstly clarify a directional relation for every drug-drug interaction. The relations can be classified into four categories such as ‘drug A enhances the action of drug B’, ‘drug A disturbs the action of drug B’, ‘drug A induces adverse effects from drug B’, or ‘unknown’. We filter out hormone-drug pairs with ‘unknown’ relation so that extracted ‘drug A - drug B interactions’ are converted to ‘influence from drug A to drug B’. Secondly, the ‘influence from drug A to drug B’ is extracted if drug A is one of the human hormones we collect from EndoNet (i.e. from ‘influence from drug A to drug B’ to ‘influence from hormone A to drug B’). We finally choose it as a gold standard sample if its drug is included in target disease-treating drugs.

### Selection of diseases

We aim to apply HIDEEP to twenty as various diseases as possible. To this end, we extract 139 diseases which have at least three gold standard samples as well as at least one disease gene. It is firstly required to filter out similar or superior diseases of other diseases to avoid overlaps. Thus, we eliminate superior diseases which have over two subordinate (child) diseases based on MeSH hierarchical trees^33^. Second, diseases are tested whether they have similar MESH descriptions or neighbor MESH tree numbers to other diseases, and then similar diseases except for one representative disease are eliminated. When extracted diseases are sorted by the sizes of gold standard samples, diseases on high ranks are mostly non-cancer diseases. In order to avoid any bias coming from particular disease types, we determine to cover cancer diseases as well as non-cancer diseases even though the cancer types are not on high ranks in sorted disease list. Consequentially fifteen non-cancer diseases and five cancer diseases are selected for case studies.

### Inference of drug effect paths

**Figure 6:**
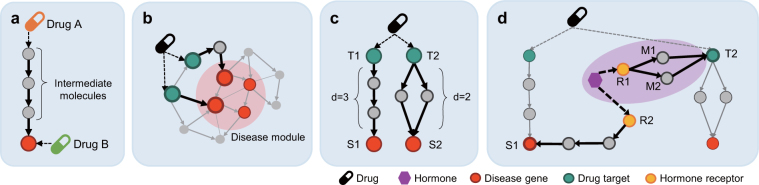
Drug effect paths and hormone effect paths. Each node type means drugs (drug symbol), hormones (purple), drug targets (green), disease genes (red), hormone targets (yellow), and other molecules (gray). (**a**) Drugs can therapeutically target disease genes in direct or indirect ways. (**b**) A drug targets subset of diseases genes clustering each other, forming a disease module (light red area). (**c**) Drug effect paths are defined as the shortest paths from each drug target to the nearest disease gene. (**d**) Hormone effect paths are defined as the very shortest paths among the shortest paths from each receptor to the nearest molecule of drug effect paths (light purple area).

In order to show therapeutic efficacy for disease treatment, drugs ultimately target disease genes either by indirectly affecting them via intermediate molecules or by directly binding to them (Fig. 6a). Disease genes tend to cluster each other by being in the neighborhood, and diseases are caused by a breakdown of a disease gene set rather than a single disease gene^34,35^. A research group figured out that it is not necessary for a drug to target all disease genes, and they defined a new method called ‘closest paths’ which implies drug MOA^36^ (Fig. 6b). We, therefore, refer to the ‘closest paths’ and newly define drug effect paths (DEPs) as a set of all possible shortest paths from each drug target to the nearest disease gene, which implies MOA of a drug. The shortest paths between two molecules are searched based on the breadth-first search algorithm. As Fig. 6c shows, for example, a drug has two drug targets, T1 and T2, and their nearest disease genes, S1 and S2 respectively. The shortest path from T1 to S1 has four molecules and path length three, and each of the shortest paths from T2 to S2 has three molecules and path length two (Fig. 6c). Thus, here the drug has totally three DEPs.

### Inference of hormone effect paths

We assume that hormones whose receptors are closer to DEPs have the higher potential to affect drug efficacy. Thus, hormone effect paths (HEPs) are newly defined as the shortest paths from hormone receptors to molecules of DEPs and HEPs possibly cause signaling crosstalk to DEPs. As Fig. 6d shows, for example, a hormone has two receptors, R1 and R2, and their nearest molecules of DEPs, T2 and S1. The length of the shortest paths from R1 to T2 is shorter (i.e. two) than that of the shortest path from R2 to S1 (i.e. three). We only consider the very shortest paths among all shortest paths from each receptor to molecules of DEPs, thus HEPs become the shortest paths from R1 to T1 (i.e. R1 → M1 → T2 and R1 → M2 → T2). DEPs consist of the shortest paths from each drug target to the nearest disease gene, which results that the DEPs have one or more different lengths (e.g. two and three) in Fig. 6c. Whereas, HEPs consist of shortest paths from overall receptors to the nearest molecules of DEPs, which results that HEPs have the only one length (e.g. two) in Fig. 6d.

### Code availability

The code and data used in the analysis are available at: https://github.com/MijinKwon/HIDEEP.

## Electronic supplementary material

Supplementary Information
