# Supplementary material for: HIDEEP: a systems approach to predict hormone impacts on drug efficacy based on effect paths
Source: Sci Rep. 2017 Nov 30;7:16600. doi: 10.1038/s41598-017-16855-8 (PMC5709390; doi:10.1038/s41598-017-16855-8)
Supplement: Supplementary file 1 — Supplementary Information [file 41598_2017_16855_MOESM1_ESM.pdf]

HIDEEP: a systems approach to predict hormone impacts on drug efficacy based on effect paths

Authors

Mijin Kwon[1], Jinmyung Jung[2,3], Hasun Yu[2], Doheon Lee[1,2,*]

[1] Department of Bio and Brain Engineering, KAIST, 291 Daehak-ro, Yuseong-gu, Daejeon, 305-701, Republic of Korea

[2] Bio-Synergy Research Center, 291 Daehak-ro, Yuseong-gu, Daejeon, 305- 701, Daejeon, Republic of Korea

[3] Department of Applied Statistics, College of Economics and Business, The University of Suwon, Bongdam-eup, Hwaseong-si, Gyeonggi-do, 18323, Republic of Korea

* Corresponding author

*Correspondence to dhlee@kaist.ac.kr

Supplementary Figures

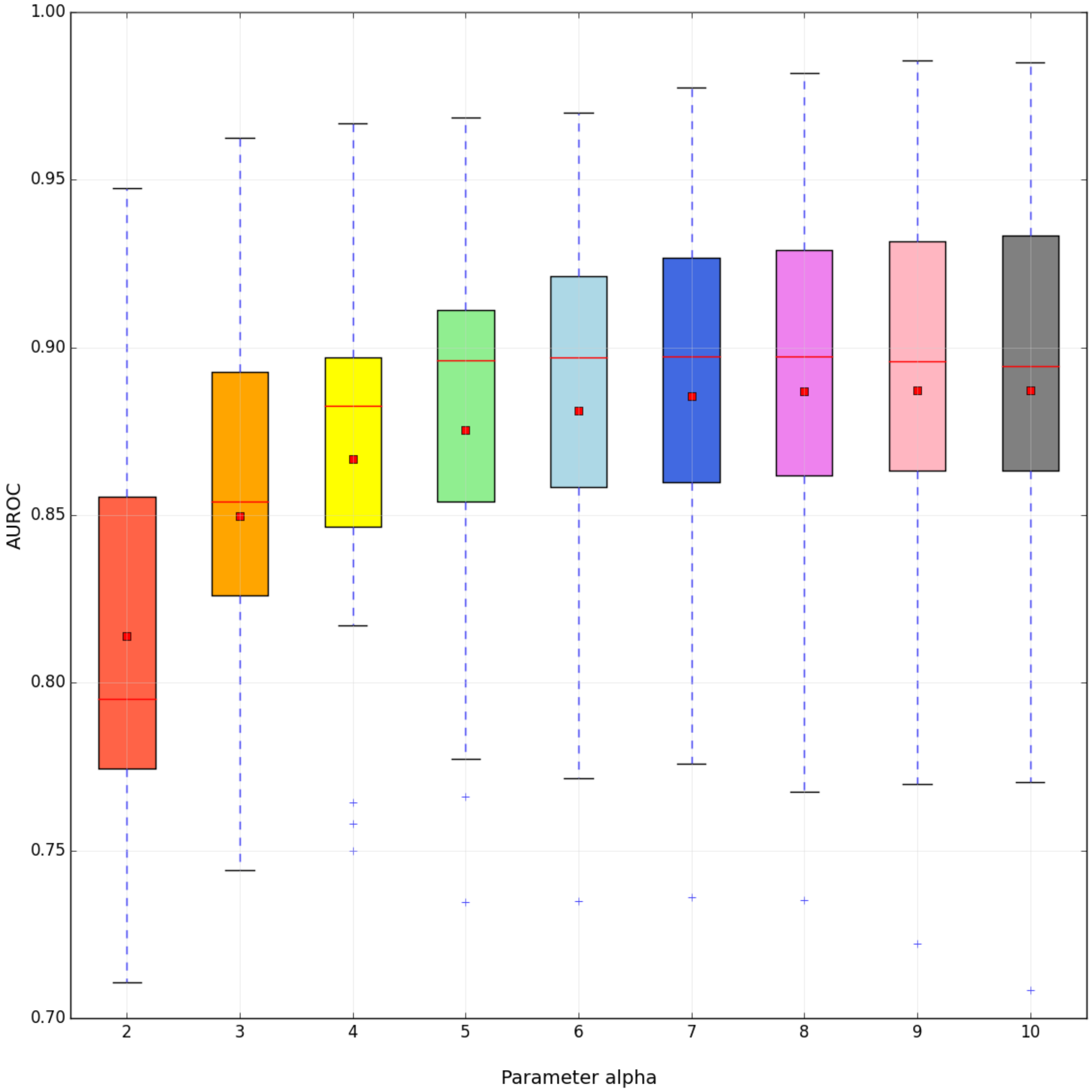

Supplementary Figure S1 Performance evaluation of the area under the receiver operating characteristic curve (AUROC) while varying a decay constant 'α'

Each box represents the distribution of AUROC values for twenty diseases given a decay constant alpha. Here, in order to cover diverse hormone-drug pairs, we use datasets consisting of gold standard sets and unlabeled sets with '10 times' of corresponding gold standard sets, among four types of sizes. A red small rectangle represents a mean value of each box. As alpha increases, mean AUROC values continuously increase as well but become saturated when α, the decay constant of the for scoring function, is 8.

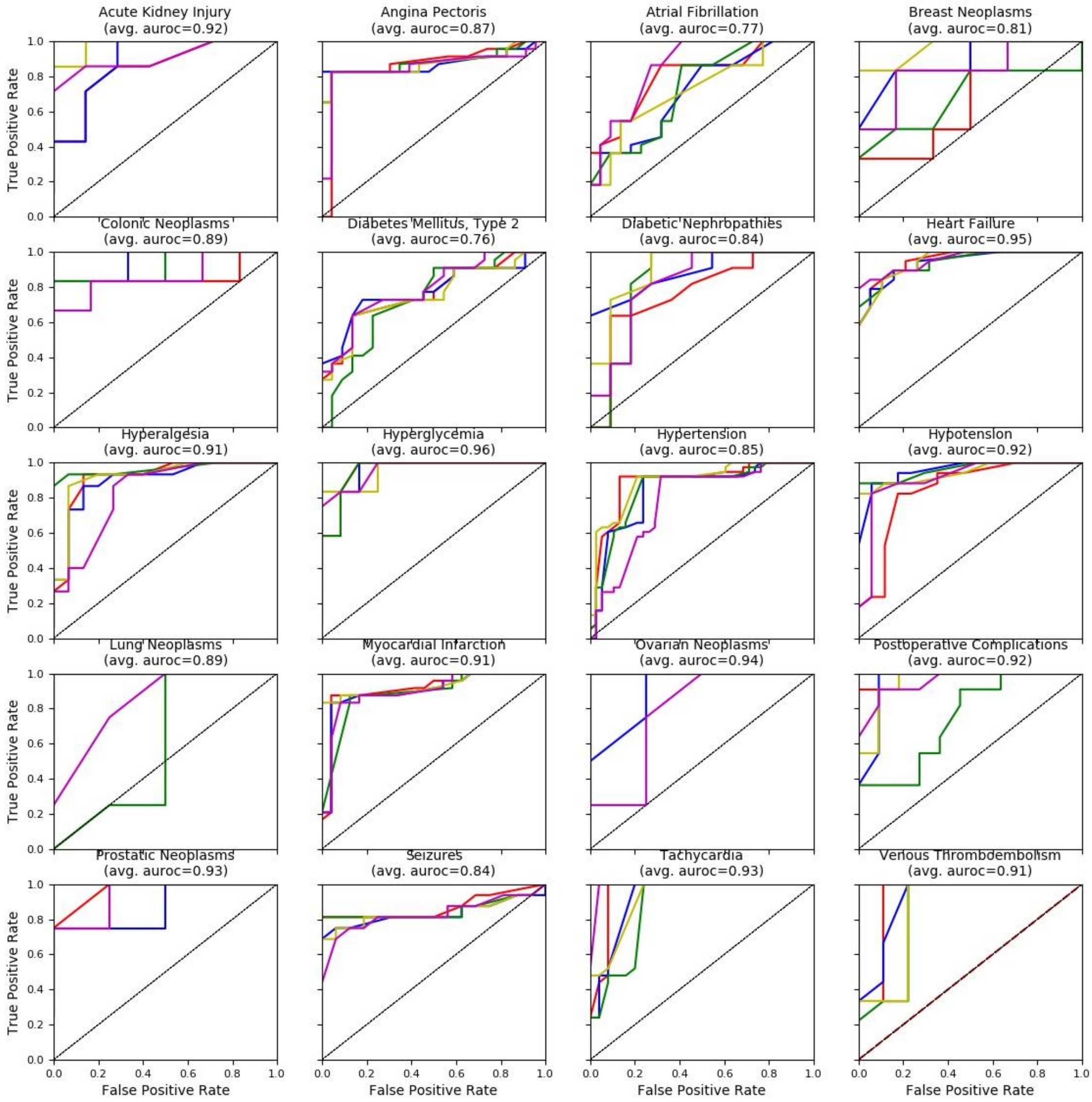

Supplementary Figure S2 Receiver Operating Characteristics (ROC) curve for five datasets whose unlabeled dataset size is 'one' time of the corresponding gold standard sets

There are 20 boxes corresponding to 20 diseases. Five ROC curves of five datasets for each disease are represented in different colors (red, green, blue, yellow green, and violet). Here five datasets consist of a gold standard set and five unlabeled sets with '1' times of the corresponding gold standard set On the top of each box, the average value of area under the ROC curves (AUROCs) for five datasets is shown.

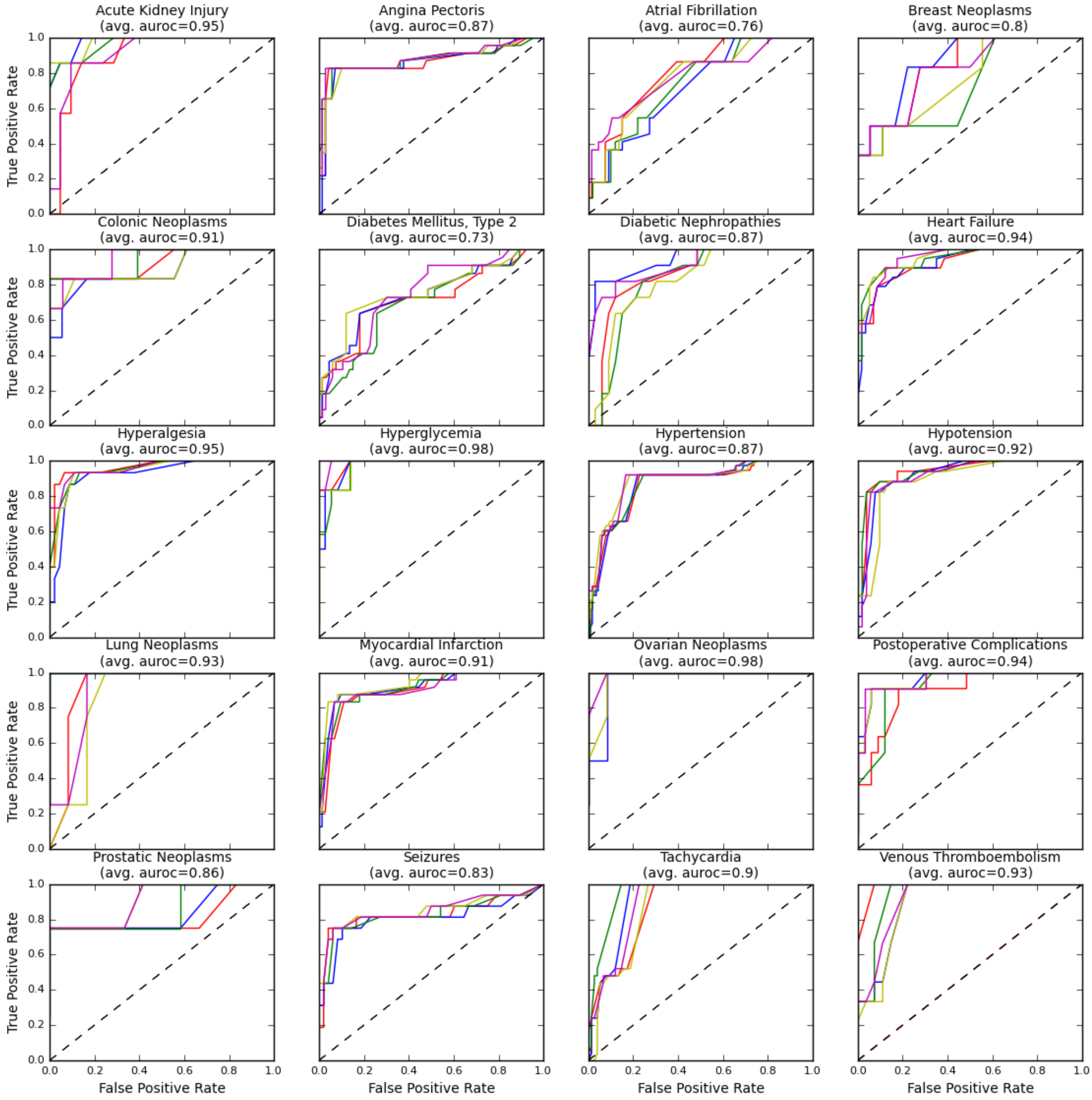

Supplementary Figure S3 Receiver Operating Characteristics (ROC) curve for five datasets whose unlabeled dataset size is ‘three’ times of the corresponding gold standard sets

There are 20 boxes corresponding to 20 diseases. Five ROC curves of five datasets for each disease are represented in different colors (red, green, blue, yellow green, and violet). Here five datasets consist of a gold standard set and five unlabeled sets with ‘3’ times of the corresponding gold standard set. On the top of each box, the average value of area under the ROC curves (AUROCs) for five datasets is shown.

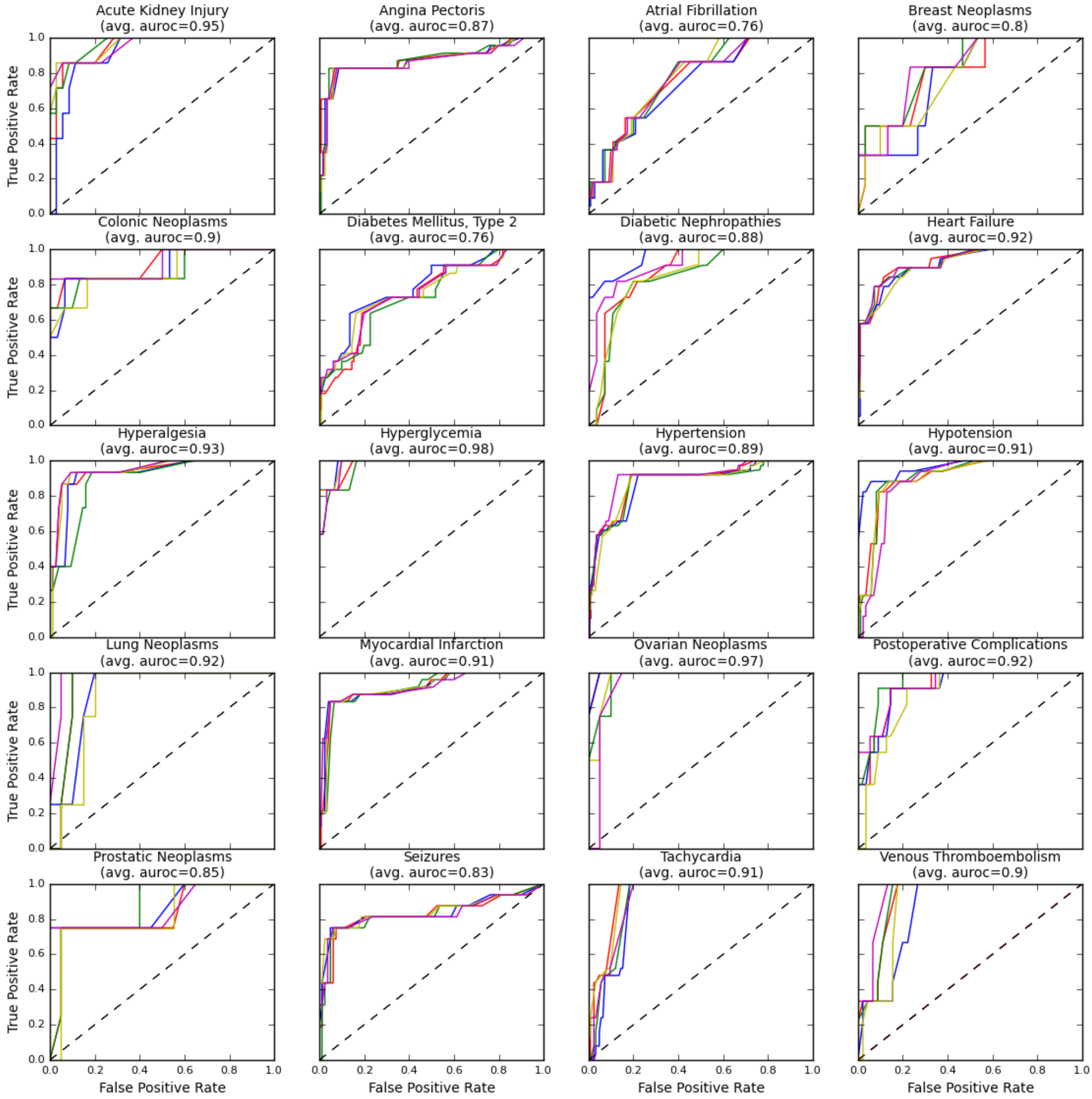

Supplementary Figure S4 Receiver Operating Characteristics (ROC) curve for five datasets whose unlabeled dataset size is 'five' times of the corresponding gold standard sets

There are 20 boxes corresponding to 20 diseases. Five ROC curves of five datasets for each disease are represented in different colors (red, green, blue, yellow green, and violet). Here five datasets consist of a gold standard set and five unlabeled sets with '5' times of the corresponding gold standard set. On the top of each box, the average value of area under the ROC curves (AUROCs) for five datasets is shown.

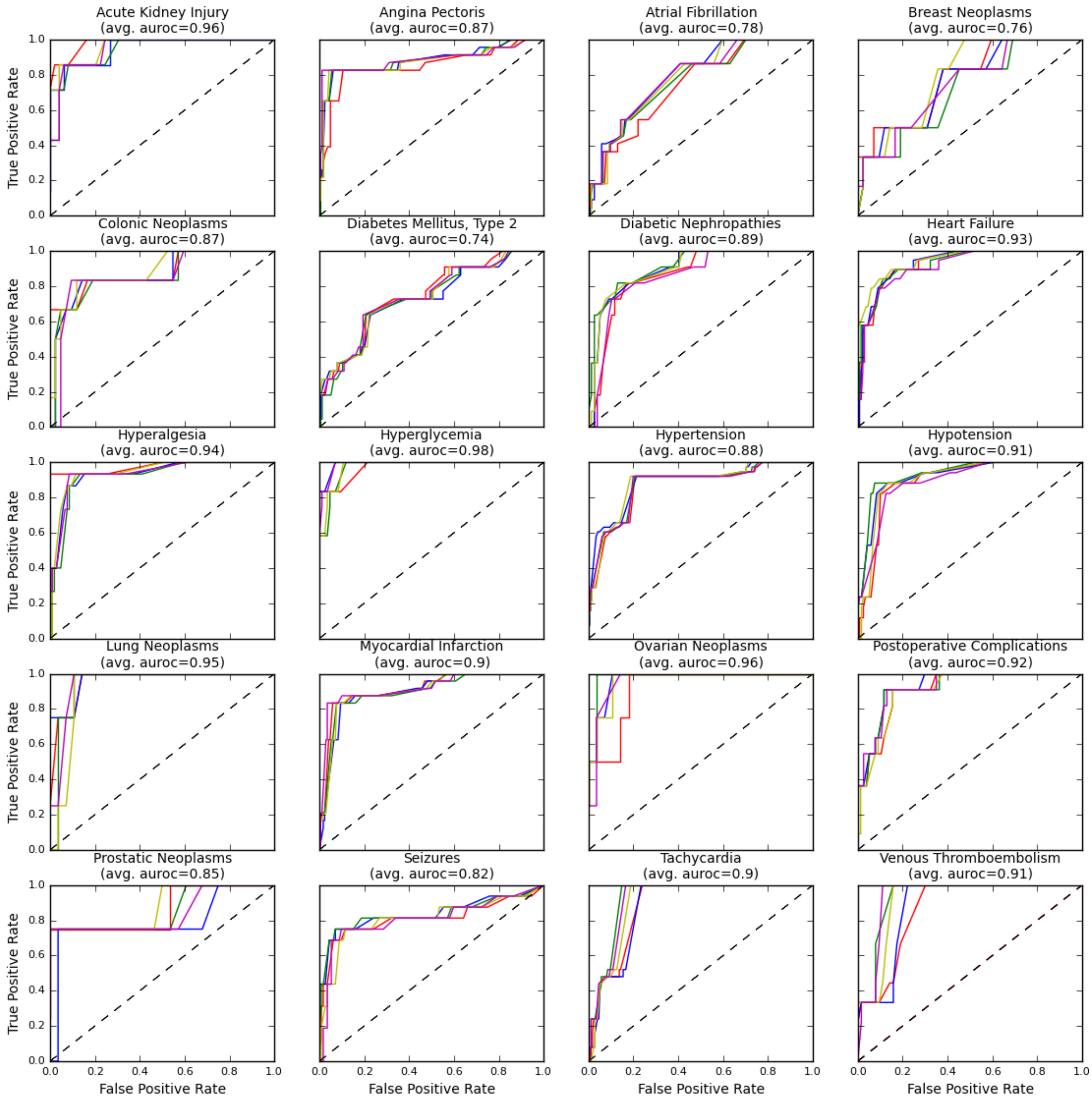

Supplementary Figure S5 Receiver Operating Characteristics (ROC) curve for five datasets whose unlabeled dataset size is 'seven' times of the corresponding gold standard sets

There are 20 boxes corresponding to 20 diseases. Five ROC curves of five datasets for each disease are represented in different colors (red, green, blue, yellow green, and violet). Here five datasets consist of a gold standard set and five unlabeled sets with '7' times of the corresponding gold standard set. On the top of each box, the average value of area under the ROC curves (AUROCs) for five datasets is shown.

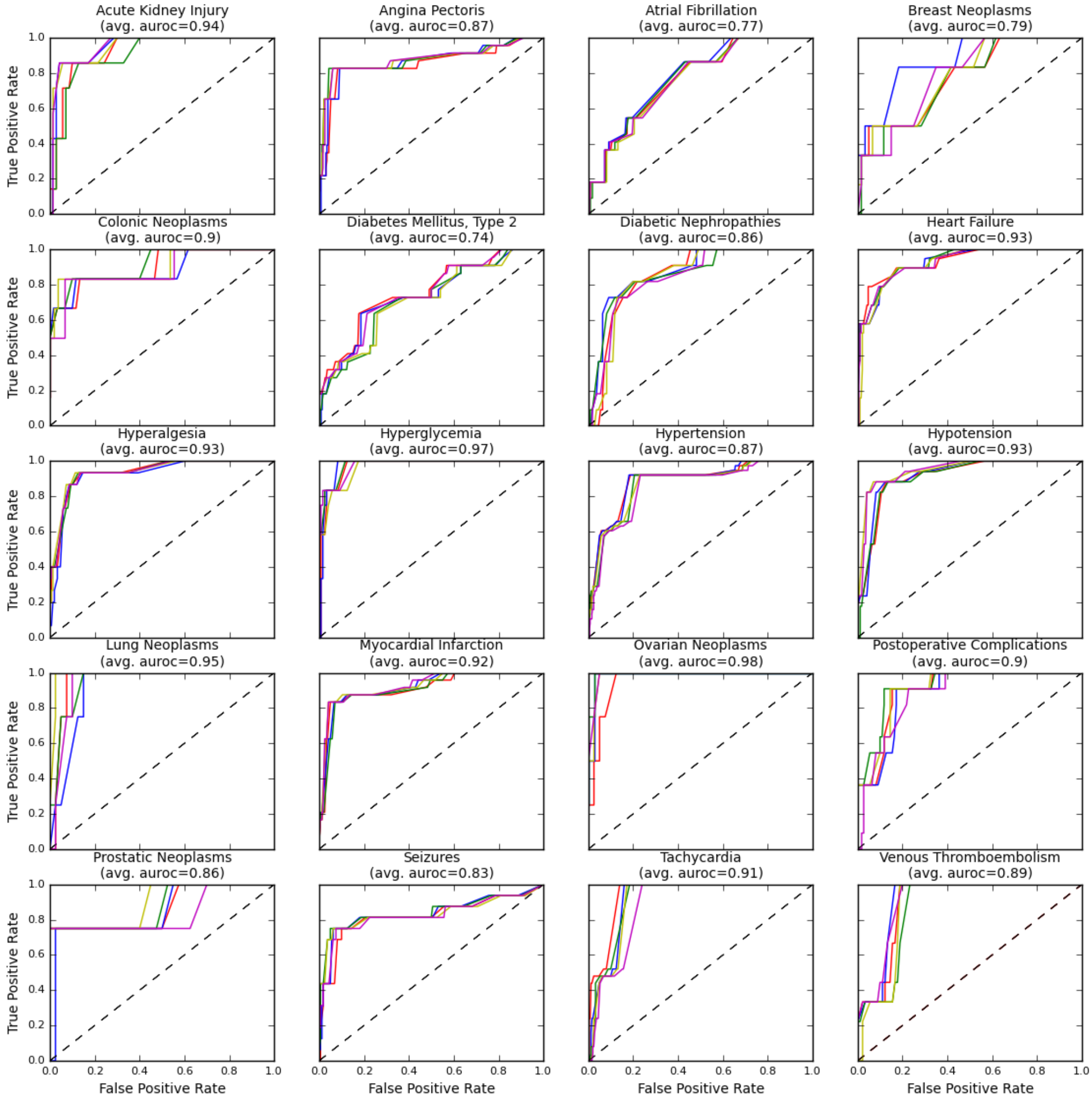

Supplementary Figure S6 Receiver Operating Characteristics (ROC) curve for five datasets whose unlabeled dataset size is ‘ten’ times of the corresponding gold standard sets

There are 20 boxes corresponding to 20 diseases. Five ROC curves of five datasets for each disease are represented in different colors (red, green, blue, yellow green, and violet). Here five datasets consist of a gold standard set and five unlabeled sets with ‘10’ times of the corresponding gold standard set. On the top of each box, the average value of area under the ROC curves (AUROCs) for five datasets is shown.

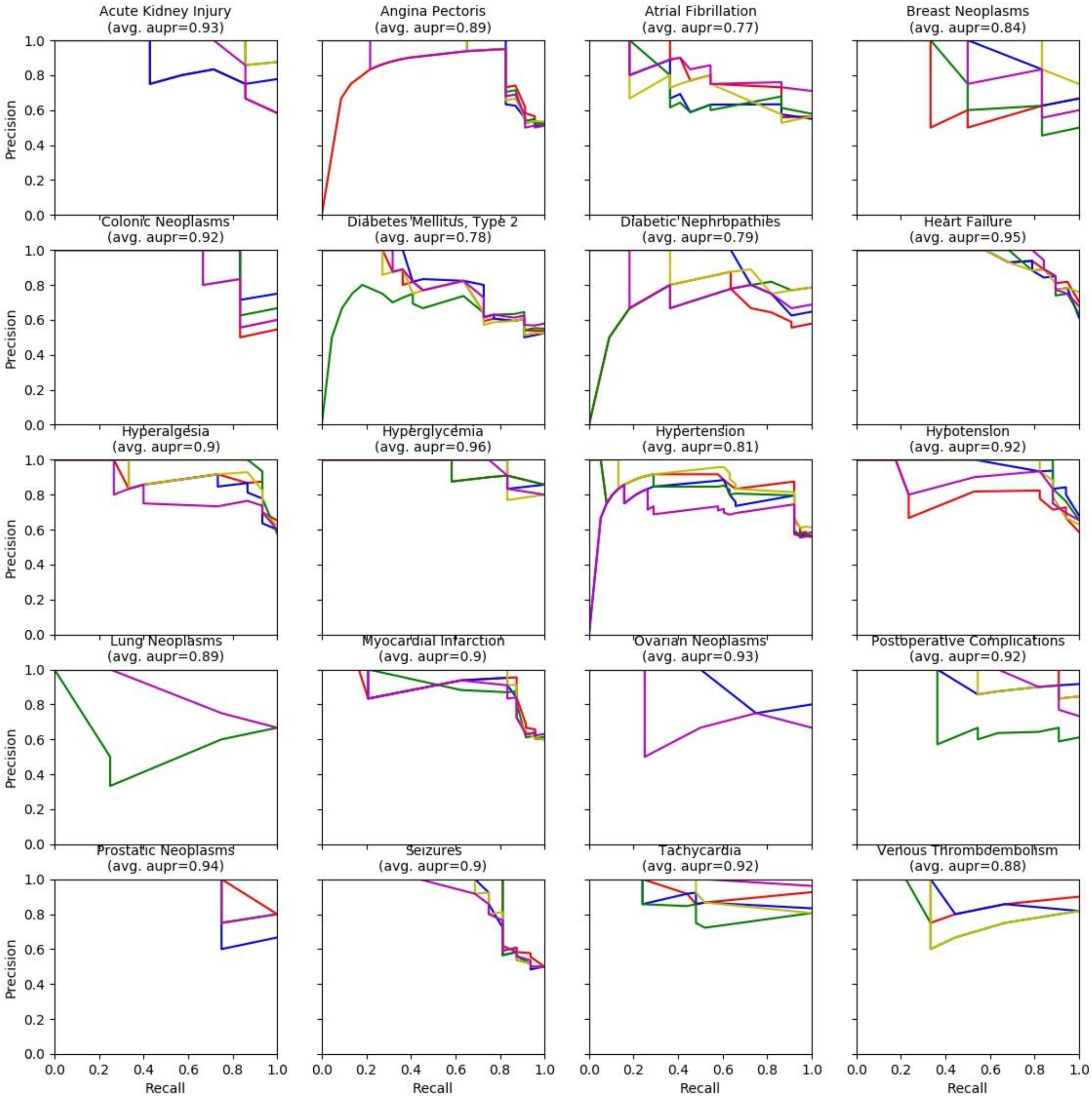

Supplementary Figure S7 Precision-recall curve for five datasets whose unlabeled dataset size is 'one' time of the corresponding gold standard sets

There are 20 boxes corresponding to 20 diseases. Five precision-recall curves of five datasets for each disease are represented in different colors (red, green, blue, yellow green, and violet). Here five datasets consist of a gold standard set and five unlabeled sets with '1' times of the corresponding gold standard set. On the top of each box, the average value of area under the precision-recall curves (AUPRs) for five datasets is shown.

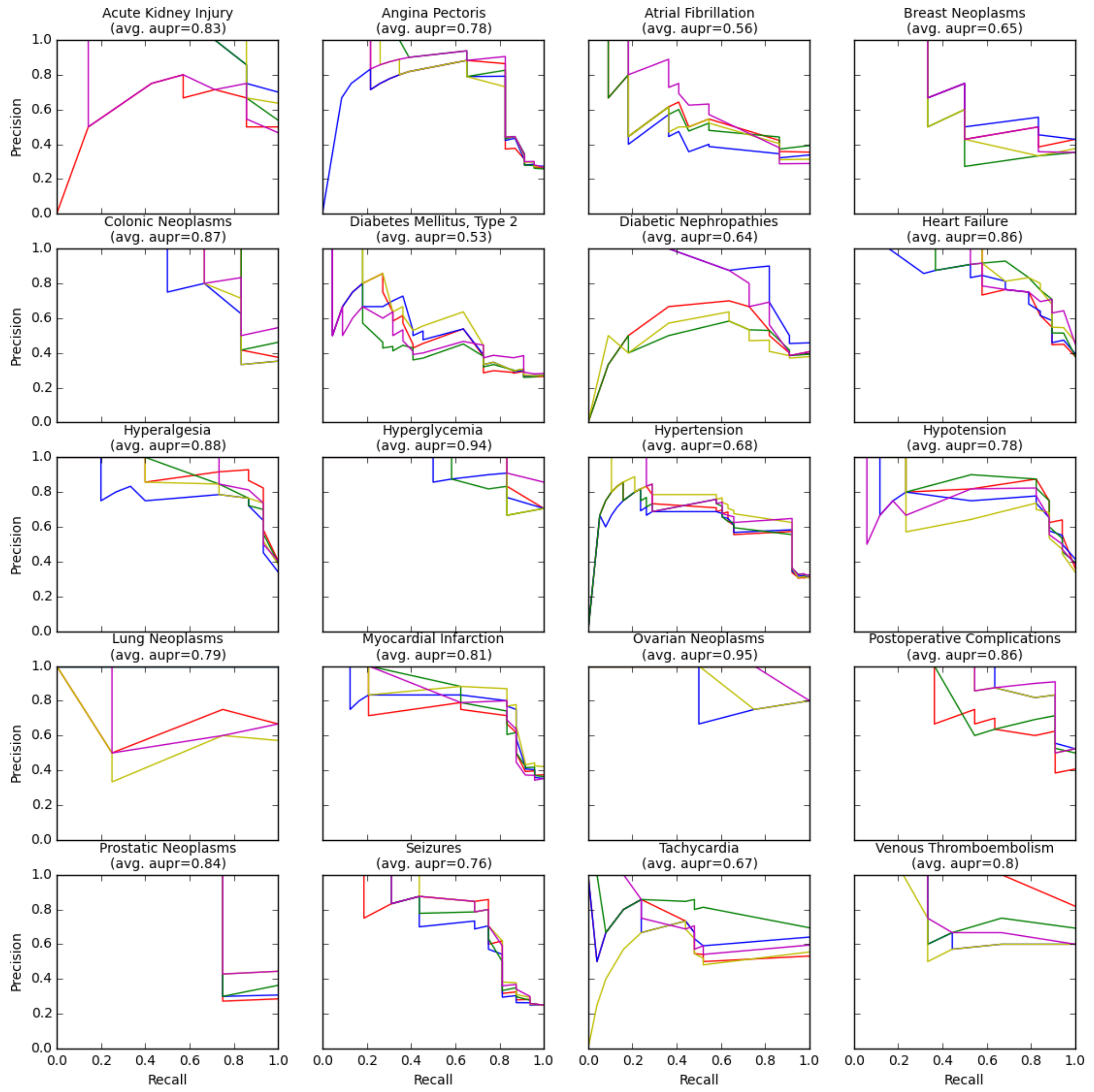

Supplementary Figure S8 Precision-recall curve for five datasets whose unlabeled dataset size is 'three' times of the corresponding gold standard sets

There are 20 boxes corresponding to 20 diseases. Five precision-recall curves of five datasets for each disease are represented in different colors (red, green, blue, yellow green, and violet). Here five datasets consist of a gold standard set and five unlabeled sets with '3' times of the corresponding gold standard set. On the top of each box, the average value of area under the precision-recall curves (AUPRs) for five datasets is shown.

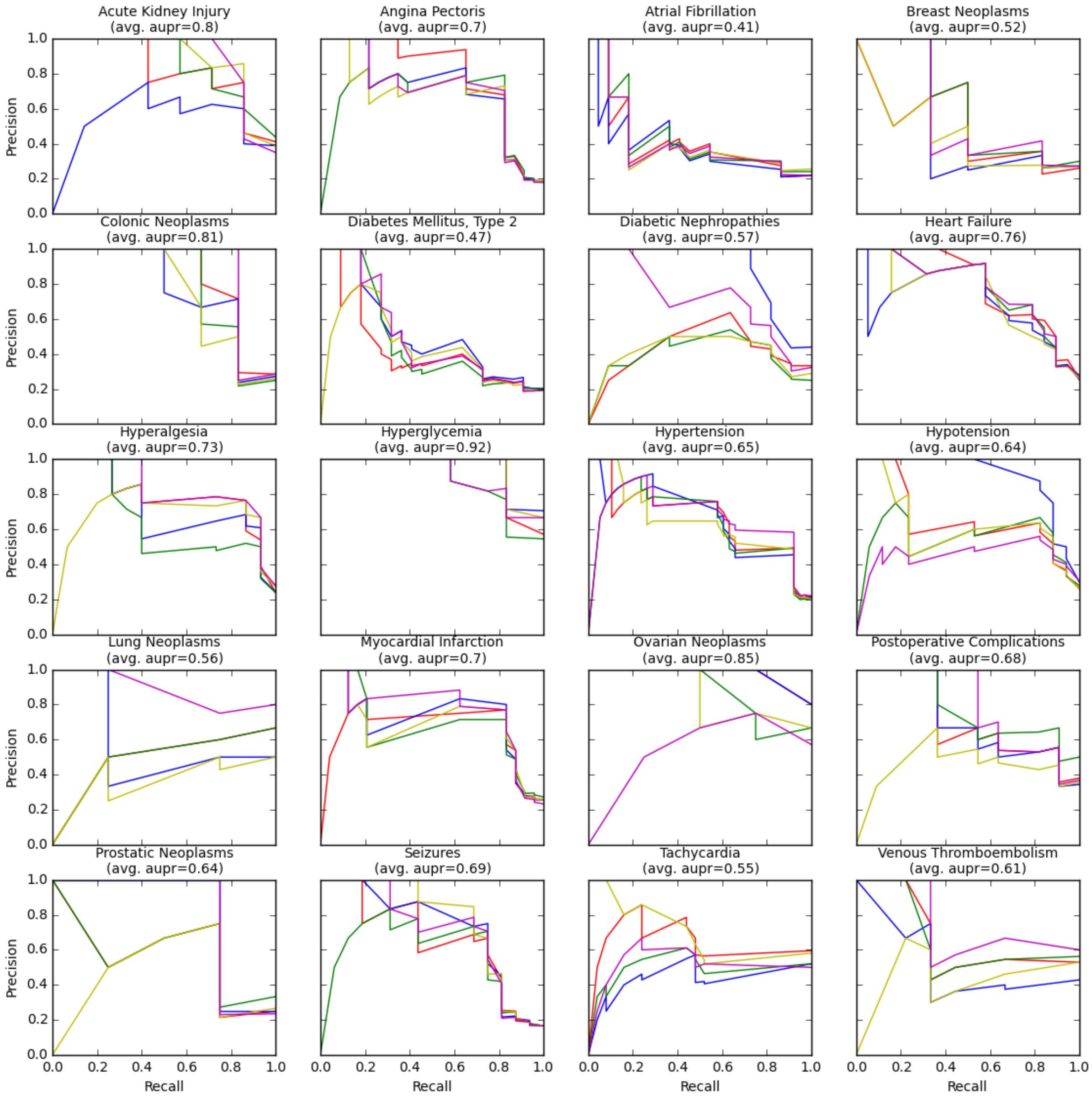

Supplementary Figure S9 Precision-recall curve for five datasets whose unlabeled dataset size is ‘five’ times of the corresponding gold standard sets

There are 20 boxes corresponding to 20 diseases. Five precision-recall curves of five datasets for each disease are represented in different colors (red, green, blue, yellow green, and violet). Here five datasets consist of a gold standard set and five unlabeled sets with ‘5’ times of the corresponding gold standard set. On the top of each box, the average value of area under the precision-recall curves (AUPRs) for five datasets is shown.

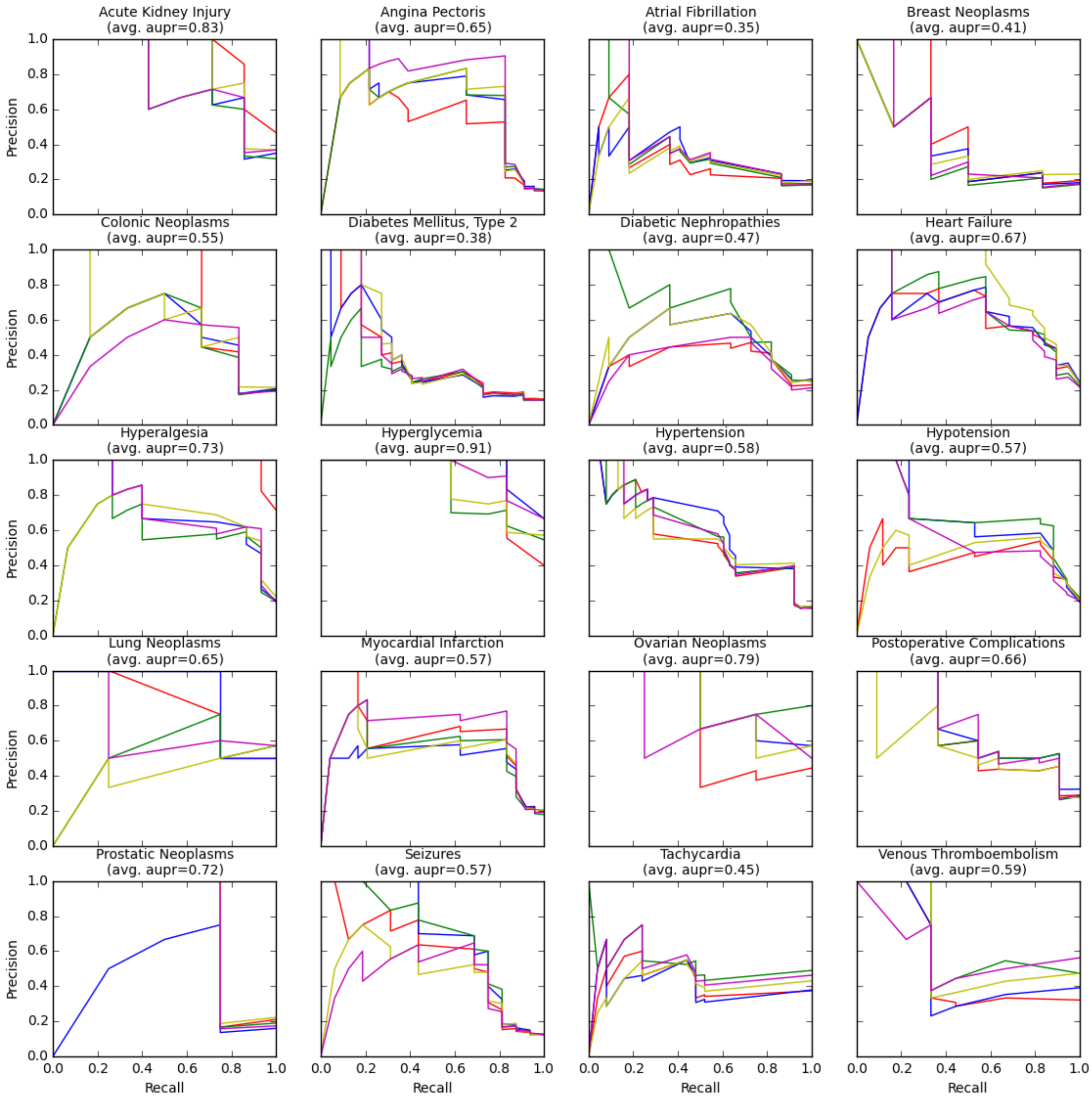

Supplementary Figure S10 Precision-recall curve for five datasets whose unlabeled dataset size is ‘seven’ times of the corresponding gold standard sets

There are 20 boxes corresponding to 20 diseases. Five precision-recall curves of five datasets for each disease are represented in different colors (red, green, blue, yellow green, and violet). Here five datasets consist of a gold standard set and five unlabeled sets with ‘7’ times of the corresponding gold standard set. On the top of each box, the average value of area under the precision-recall curves (AUPRs) for five datasets is shown.

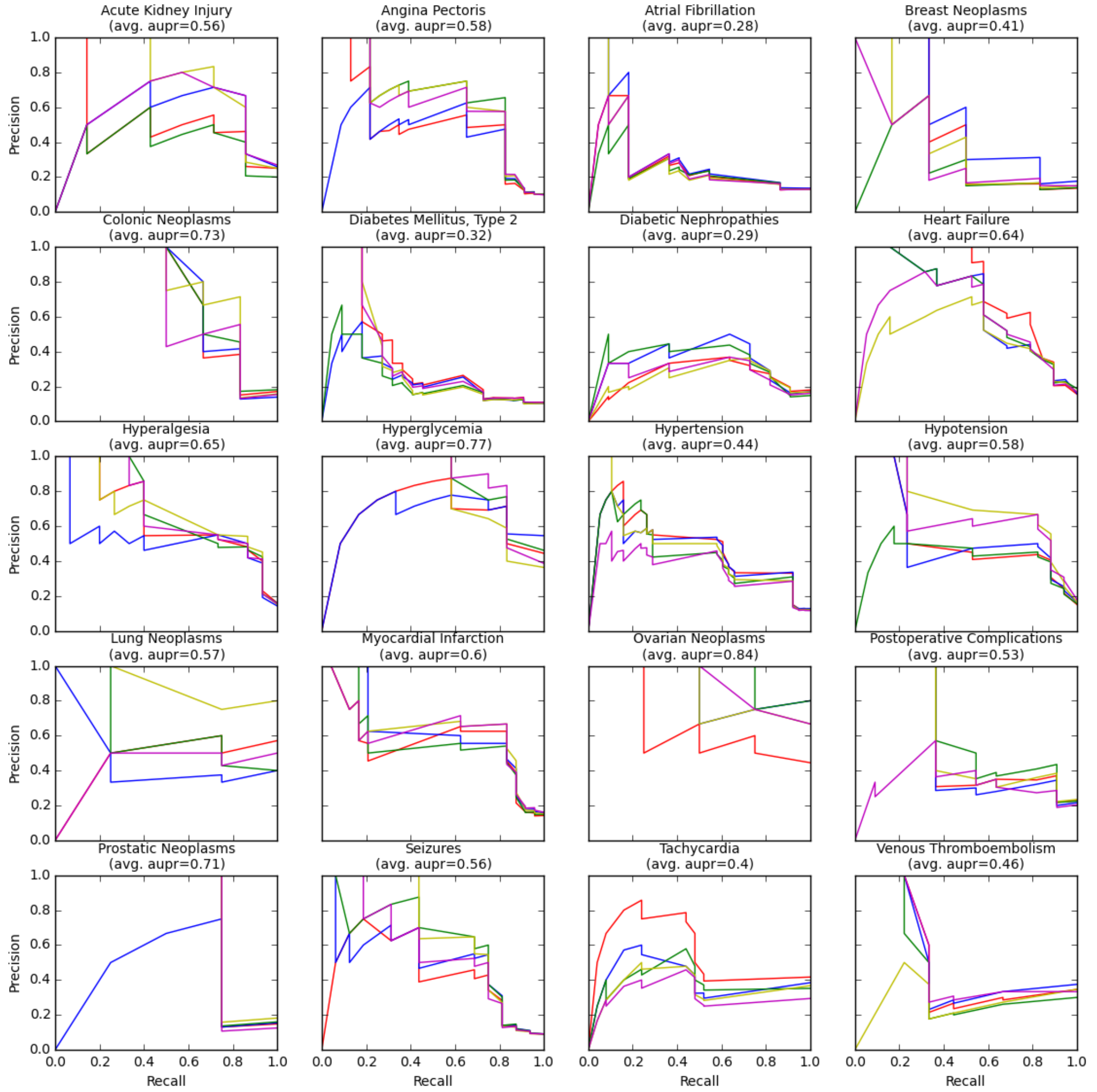

Supplementary Figure S11 Precision-recall curve for five datasets whose unlabeled dataset size is 'ten' times of the corresponding gold standard sets

There are 20 boxes corresponding to 20 diseases. Five precision-recall curves of five datasets for each disease are represented in different colors (red, green, blue, yellow green, and violet). Here five datasets consist of a gold standard set and five unlabeled sets with '10' times of the corresponding gold standard set. On the top of each box, the average value of area under the precision-recall curves (AUPRs) for five datasets is shown.

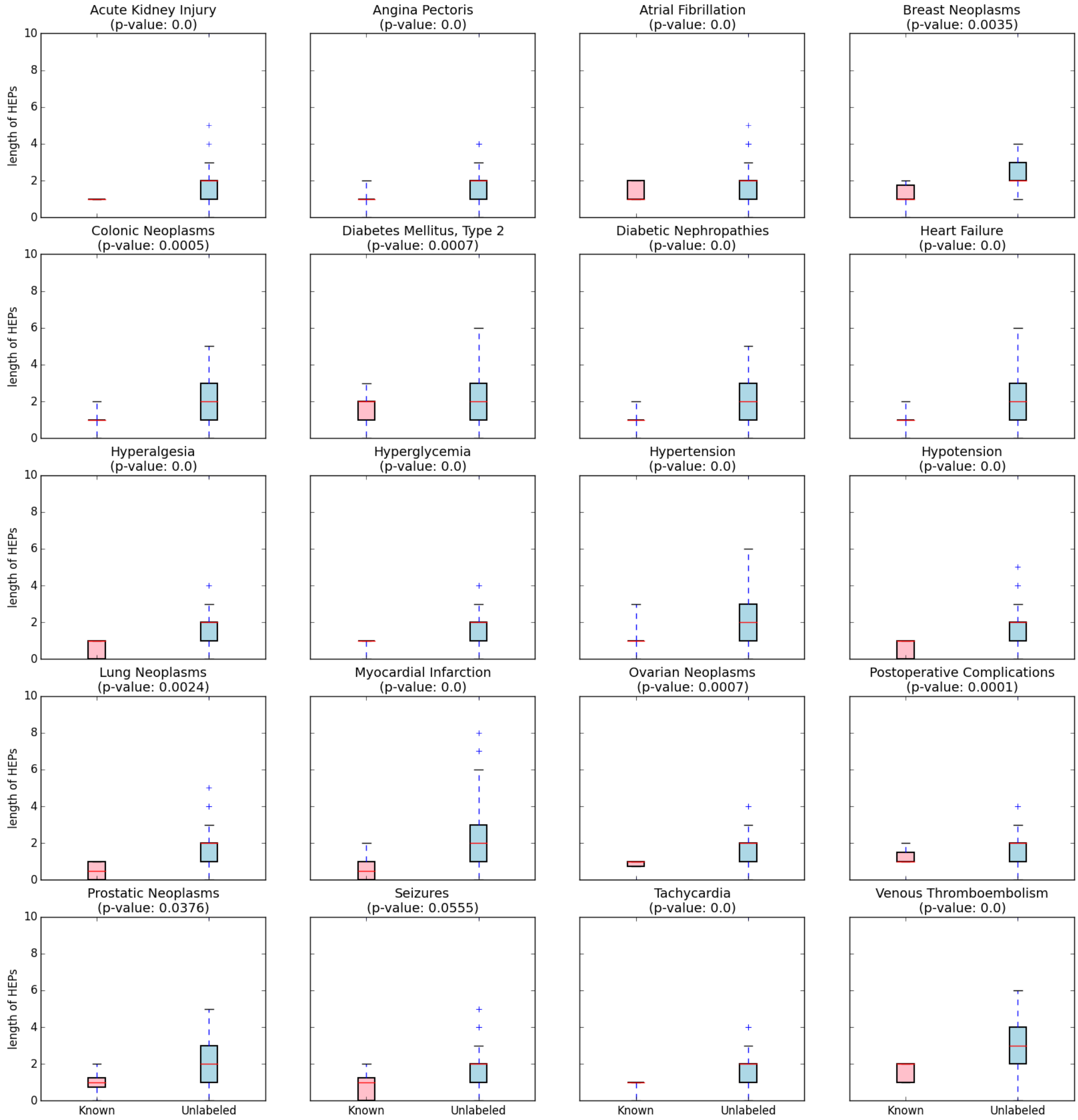

Supplementary Figure S12 Distributions of hormone effect paths (HEP) lengths for gold standard sets and unlabeled sets

Each box represents distributions of a gold standard set (pink) and five unlabeled sets ('10 times' size of the corresponding gold standard set) (light blue). We implemented a statistical multivariate t-test to assess the significance of the difference between the mean lengths of hormone effect paths (HEP) of two kinds of independent sample sets (i.e. gold standard sets and unlabeled sets).

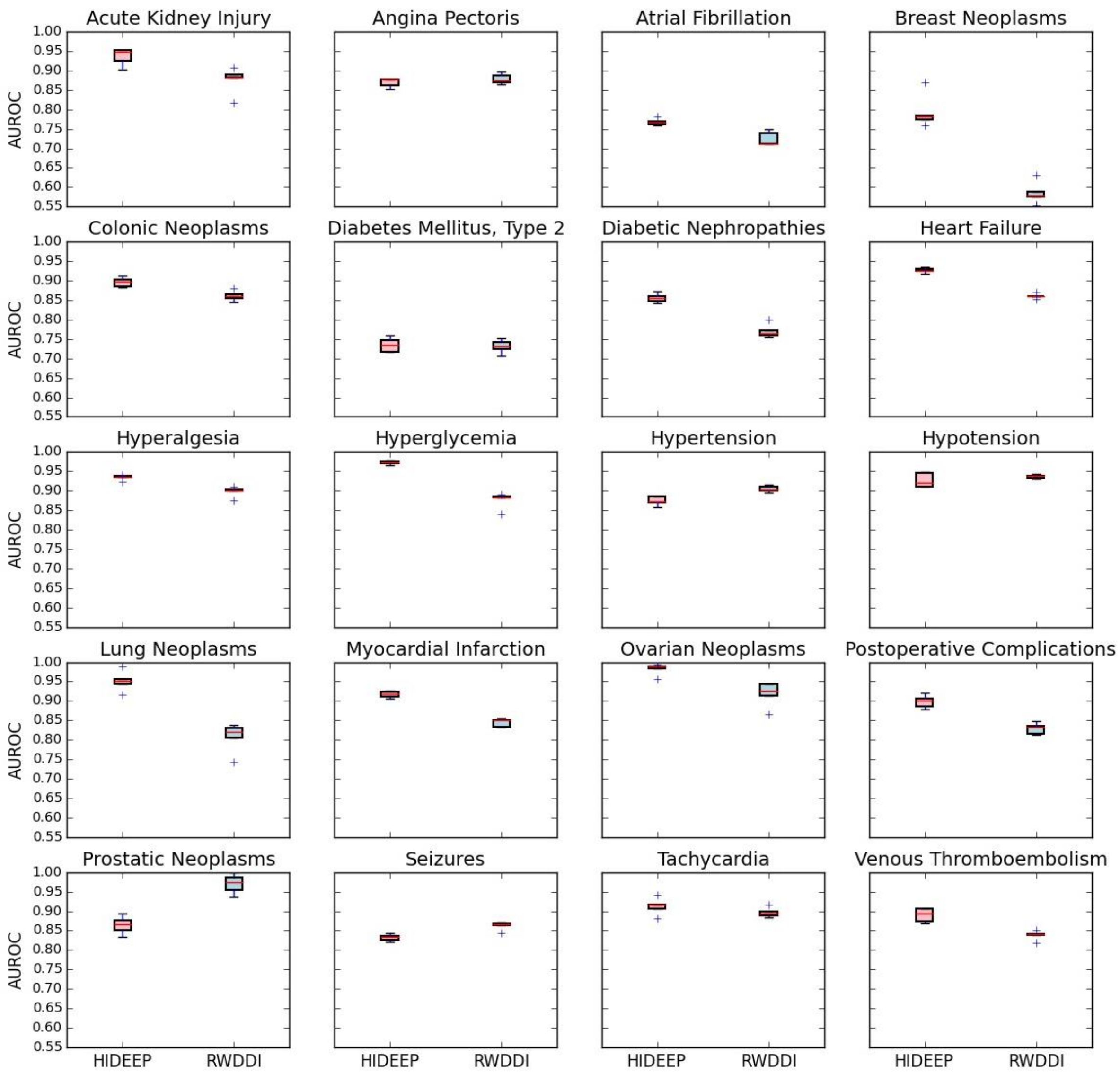

Supplementary Figure S13 Performance comparison with a previous study (RWDDI)

The performance of HIDEEP (pink) and RWDDI (light blue) for twenty diseases was evaluated by the area under the receiver operating characteristic curve (AUROC). For this performance comparison, both models are applied to five datasets consisting of a gold standard set and five unlabeled sets with '10 times' for each disease. Thus, each box includes five AUROC values for five datasets.

Supplementary Tables

Supplementary Table 1 The number of gold standard samples and unlabeled samples with five different sizes

Disease	# of gold standard samples	# of unlabeled samples				
		1 times	3 times	5 times	7 times	10 times
Hypertension	38	38	114	190	266	380
Tachycardia	25	25	75	125	175	250
Myocardial Infarction	24	24	72	120	168	240
Angina Pectoris	23	23	69	115	161	230
Diabetes Mellitus, Type 2	22	22	66	110	154	220
Atrial Fibrillation	22	22	66	110	154	220
Heart Failure	19	19	57	95	133	190
Hypotension	17	17	51	85	119	170
Seizures	15	15	45	75	105	150
Hyperalgesia	15	15	45	75	105	150
Hyperglycemia	12	12	36	60	84	120
Postoperative Complications	11	11	33	55	77	110
Diabetic Nephropathies	11	11	33	55	77	110
Venous Thromboembolism	9	9	27	45	63	90
Acute Kidney Injury	7	7	21	35	49	70
Breast Neoplasms	6	6	18	30	42	60
Colonic Neoplasms	6	6	18	30	42	60
Ovarian Neoplasms	4	4	12	20	28	40
Lung Neoplasms	4	4	12	20	28	40
Prostatic Neoplasms	4	4	12	20	28	40

Supplementary Table 2 A two-sample multivariate t-test results for hormone effect path (HEP) lengths of a gold standard set and an unlabeled set

Disease	t-statistic	p-value
Acute Kidney Injury	-10.00419065	6.98E-21
Angina Pectoris	-8.419111078	4.80E-10
Atrial Fibrillation	-6.247576479	2.08E-07
Breast Neoplasms	-4.357457049	0.00347582
Colonic Neoplasms	-5.641224508	0.000480647
Diabetes Mellitus, Type 2	-3.845133966	0.000708701
Diabetic Nephropathies	-6.723309563	4.27E-06
Heart Failure	-10.98067688	1.29E-12
Hyperalgesia	-11.01855851	9.58E-12
Hyperglycemia	-10.09176596	1.43E-13
Hypertension	-14.34399293	1.24E-20
Hypotension	-10.82515584	4.58E-12
Lung Neoplasms	-5.544637117	0.002350051
Myocardial Infarction	-11.04272973	1.12E-12
Ovarian Neoplasms	-5.712186997	0.000708068
Postoperative Complications	-5.146029738	0.000112613
Prostatic Neoplasms	-3.193228739	0.03755567
Seizures	-2.068675588	0.055517263
Tachycardia	-14.8421274	4.88E-22
Venous Thromboembolism	-8.09211323	1.42E-06

Supplementary Table 3 Cross-validation based performance evaluation of the HIDEEP at the different alpha, α

Disease	AUROC								
	**2**	**3**	**4**	**5**	**6**	**7**	**8**	**9**	**10**
Angina Pectoris	0.7976	0.8298	0.8459	0.8549	0.8622	0.8668	0.8688	0.8666	0.8636
Atrial Fibrillation	0.7334	0.7477	0.7565	0.7649	0.7710	0.7754	0.7666	0.7686	0.7695
Diabetes Mellitus, Type 2	0.7560	0.7517	0.7499	0.7354	0.7359	0.7371	0.7361	0.7231	0.7095
Diabetic Nephropathies	0.7942	0.8280	0.8477	0.8502	0.8495	0.8529	0.8554	0.8581	0.8609
Heart Failure	0.8819	0.9078	0.9201	0.9224	0.9246	0.9264	0.9277	0.9244	0.9219
Hyperalgesia	0.7850	0.8533	0.8916	0.9112	0.9231	0.9323	0.9362	0.9409	0.9436
Hyperglycemia	0.9471	0.9624	0.9676	0.9694	0.9710	0.9727	0.9738	0.9749	0.9744
Hypertension	0.7917	0.8251	0.8467	0.8572	0.8648	0.8704	0.8736	0.8761	0.8782
Hypotension	0.7931	0.8605	0.8932	0.9104	0.9177	0.9228	0.9273	0.9299	0.9312
Myocardial Infarction	0.7913	0.8610	0.8943	0.9137	0.9183	0.9188	0.9180	0.9168	0.9182
Postoperative Complications	0.8601	0.8941	0.9028	0.9003	0.9031	0.9032	0.9000	0.8945	0.8871
Seizures	0.7532	0.8012	0.8168	0.8213	0.8267	0.8285	0.8303	0.8324	0.8336
Tachycardia	0.8025	0.8554	0.8812	0.8948	0.9039	0.9099	0.9130	0.9148	0.9163
**Average**	**0.8067**	**0.8445**	**0.8627**	**0.8697**	**0.8747**	**0.8782**	**0.8790**	**0.8785**	**0.8775**
